# Gender differences in marital violence: A cross-ethnic study among Bengali, Garo, and Santal communities in rural Bangladesh

**DOI:** 10.1371/journal.pone.0251574

**Published:** 2021-05-19

**Authors:** Rabiul Karim, Hafijur Rahman, Suchona Rahman, Tanzima Zohra Habib, Katarina Swahnberg

**Affiliations:** 1 Department of Social Work, University of Rajshahi, Rajshahi, Bangladesh; 2 Department of Health and Caring Sciences, Faculty of Health and Life Sciences, Linnaeus University, Kalmar, Sweden; Purdue University, UNITED STATES

## Abstract

Studies on marital violence (MV) in Bangladesh have primarily focused on the women of the mainstream *Bengali* people, although half of the population is men, and there are also ethnic minority communities with diverse gender constructions. The current study examined the gender differences in MV among the matrilineal ethnic minority *Garo*, patrilineal ethnic minority *Santal*, and the patrilineal mainstream *Bengali* communities in rural Bangladesh. Adopting a cross-sectional design, we randomly included 1,929 currently married men and women from 24 villages. We used cross-tabulations as well as multivariate logistic regressions to estimate the ethnic and gender differences in MV. Data revealed that women were widely exposed to different types of MV, while only a few men experienced such abuses. It showed that 95.6% of the women experienced emotional abuse, 63.5% physical abuse, 71.4% sexual abuse, and 50.6% poly-victimization, whereas these rates were quite low among the men (emotional = 9.7%, physical = 0.7%, sexual = 0.1%). No men reported poly-victimization. The odds ratio (OR) for emotional, physical, and sexual MV were respectively, 184.44 (95% CI = 93.65−363.24, *p*<0.001), 449.23 (95% CI = 181.59−1111.35, *p*<0.001), and 2789.71(95% CI = 381.36−20407.08, *p*<0.001) for women compared to men. Data further revealed that matrilineal *Garo* women experienced less MV (emotional = 90.7%, physical = 53.4%, sexual = 64.0%, poly = 38.8%) than the patrilineal *Santal* (emotional = 99.4%, physical = 67.3%, sexual = 71.3%, poly = 53.9%) and *Bengali* women (emotional = 96.6%, physical = 69.6%, sexual = 78.8%, poly = 58.9%). Multivariate regressions also showed that the *Bengali* society perpetrated more physical (*OR* = 1.90, 95% CI = 1.27−2.85, *p* = 0.002) and sexual (*OR* = 2.04, 95% CI = 1.34−3.10, *p* = 0.001) MV than the *Garo* society. It appears that MV is largely a gendered issue in the country. Though both women and men can be the victims of MV, the nature/extent of victimization noticeably differs according to the social organization. Matrilineal society appears to be less abusive than the patrilineal one. Interventions aimed to prevent domestic violence in rural Bangladesh should take these findings into account.

## Introduction

Marital violence (MV) accounts for the highest percentage among all types of domestic violence worldwide [1, 2]. As the most common form of violence within intimate relationships, MV refers to any abusive behavior by a spouse that results in physical, psychological, or sexual harm or suffering to another spouse [3]. MV against women is a global public health issue [1]. It is widely prevalent in many low- and lower-middle-income countries [4]. Women have usually been considered the primary victims of MV. Previous studies worldwide have primarily focused on female victims and viewed men as the perpetrators [1, 5]. However, there is a claim that men can also be the victims of MV perpetrated by their female partners [6, 7]. Some scholars argue that both men and women can be perpetrators and victims of MV [8, 9], although the nature of victimization might be different depending on the gender of the perpetrators/victims [2, 10].

Global literature on MV is typically overwhelmed by a focus on female victims [4, 11]. The gender-disaggregated analysis on MV is still rare worldwide; however, it is important to understand the phenomenon from a holistic perspective. Such a perspective emphasizes the understanding of MV, incorporating its different spectrum, including a need-assessment related to identifying MV acts and their perpetrators/victims [12]. Traditionally, literature on MV suggests only one type of victim (e.g., MV against women–men’s patriarchal controlling over their wives)–which is not only an oversimplification of the complex social phenomenon, but it also excludes the needs of victims from male, and homosexual groups [12]. Thus, in order to prevent MV, it is crucial to know how men and women are exposed to MV. However, there is a lack of studies on the issue, particularly involving low- and lower-middle-income countries.

Many societies support or tolerate husbands’ abusive behaviors against wives [13]. Thus, MV is viewed as a gendered issue prevalent in a patriarchal society [14]. A few scholars also believe that men are physically stronger than women; hence, they are less likely to be the victims of abuse by a female partner [6]. Although studies devoted to wife perpetrated violence against men are lacking, numerous studies on spousal abuse, primarily conducted in developed countries, revealed that both men and women could perpetrate violence against their partner [6–8, 15]. Even though feminist scholars criticized the idea that husbands could also be the victims of MV [16, 17], researchers revealed that husbands resort to violence to dominate their wives, whereas both women and men can perpetrate MV against their intimate partner as a result of the escalation of marital conflict [2, 10]. Thus, it would not be wise to exclude men as being victims of MV. Both women and men should be included in such studies as potential victims of MV.

As a human issue, MV should be viewed from both the male and female perspectives [6]. However, men have not yet been included as victims of MV in studies conducted in Bangladesh. In contrast, women’s exposure to MV is well depicted in the previous literature. Many studies have examined both female (as victims) and male (as perpetrators) experiences of MV [18, 19–21]. MV against women is a severe public health issue in Bangladesh, where nearly eight in ten women experience lifetime physical and/or sexual abuse from their spouse [22]. Nonetheless, no studies estimated the rates of both men’s and women’s exposure to MV in the country.

Previous studies have also not incorporated the ethnic minority communities living in the country, while it is often assumed that MV is less common among them, particularly among the matrilineal communities [13, 23]. Previous studies offered little evidence in elucidating how the socio-cultural differences among the ethnic minority and mainstream communities may influence men’s and women’s experiences of MV. There is a paucity of studies on MV among the ethnic minorities in Bangladesh, although there are at least 27 ethnic minority communities in the country [24]. Previous population-based studies have mainly focused on the *Bengali* female population. Due to their small size in numbers, ethnic minorities are not likely to be included in the population-based sample in adequate numbers to enable separate reporting. Thus, it is also important to focus on minority communities to study the ethnic feature of MV in Bangladesh.

Bangladesh is a lower-middle-income country with a population of around 163 million. Nearly 24.3% of them fall below the poverty line–earning less than 1.90 USD a day [25]. The *Bengali* ethnolinguistic community includes 98% of the total population, whereas the ethnic minorities comprise the remaining 2%. Most ethnic minorities, e.g., Chakma, Manipuri, Santal, and so on, are patriarchal, though there are also matrilineal societies such as the Garo and Khasi. Most of them live in rural areas. Approximately 63% of the population lives in rural areas [26].

Ethnic minority communities in Bangladesh represent diverse socio-cultural features (see Table 1). The gender construction among the ethnic minorities is generally different from the mainstream *Bengali* community. It is believed that the status of women (including access to income, mobility, and freedom) is relatively high among the *Garo* and *Santal* than the Bengali (see Table 1). Thus, it is important to assess both women’s and men’s experiences of MV among different ethnic groups since the traits of MV may vary across the ethnic communities [27]. This study looks at gender-specific MV in the matrilineal ethnic *Garo*, patrilineal ethnic *Santal*, and the patriarchal mainstream *Bengali* communities in rural Bangladesh. By providing cross-ethnic and gender-specific evidence, the findings may facilitate strategies to prevent MV in the country.

**Table 1:** Women’s status in the ethnic minority *Garo* and *Santal* and mainstream *Bengali* communities in rural Bangladesh.

The Garo	The Santal	The Bengali
• One of the largest ethnic minority groups consisting of more than 200,000 people.	• A large indigenous ethnic minority community consisting of more than 250,000 people.	• The mainstream community in Bangladesh. They consist of 98% of the population.
• *Garo* people speak *Garo* (a Sino-Tibetan language). They also understand *Bengali*.	• *Santals* speak *Santali* (an Austro-Asiatic language). They also speak *Bengali*.	• They speak Bengali (an Indo-Aryan language); the country’s widely spoken language.
• Living in the northeastern parts of the county.	• Living in the northwestern parts of the country.	• The population is spread out over the country.
• Sometimes described as a matriarchal society.	• A patriarchal society–male power is obvious.	• The Bengali society is traditionally patriarchal.
• Women own all of the household properties.	• Men own most of the household properties.	• Men own most of the household properties.
• Family properties pass down to the women.	• Family properties mainly pass down to the men.	• Family properties mainly pass down to the men.
• Women are the heads of the household.	• Men are treated as the heads of the household.	• Generally, men are the heads of the household.
• Women enjoy a higher status in the family.	• The status of women is low compared to men.	• Women are regarded as ‘dependent members.’
• Both women and men are financial providers.	• Both women and men are financial providers. Women play a vital role in earning a livelihood.	• Men are expected to be the financial providers.
• Women generally perform the home-making chores, take care of the children and others.	Women also perform all home-making chores.	• Women are expected to perform all of the home-making chores, take care of the children/others.
• *Garos* have mostly converted to Christianity.	• They have mostly converted to Christianity.	• Most of them are Muslim and a few are Hindus.
• Garo society is divided into thirteen clans.	• Santal society is divided into twelve clans.	• There are two castes among the *Bengali* Muslims.
• Inter-clan marriage is strongly discouraged.	• Inter-clan marriage is strictly forbidden.	• Inter-caste marriage is no longer discouraged.
• Women have the privilege to choose a partner.	• Women and men are free to choose a partner.	• Men play the main role in partner selection.
• The husband moves to the wife’s house.	• The wife moves to the husband’s house.	• The wife moves to the husband’s house.
• The wife is highly respected in the family.	• The husband is highly respected in the family.	• The wife is expected to obey her husband.
• Descent is matrilineal.	• Descent is patrilineal.	• Descent is patrilineal.
• Physical mobility of women is high in society. Women can move freely out of the home.	• Physical mobility of women is high in society. Women can move freely out of the home.	• Veiled seclusion among women–they are not allowed to move freely out of the home vicinity.

Source: Field notes of the research team members and other sources were used to prepare the table [13, 28, 29].

## Aims and research questions

The current study estimates the ethnic and gender differences in emotional, physical, and sexual MV among the ethnic minority *Garo* and *Santal* and mainstream *Bengali* communities in rural Bangladesh. Regarding the academic debate on the gender differences/symmetry in MV, we seek to answer two research questions: (a) whether MV is a gendered issue or a natural human behavior in rural Bangladesh and (b) how MV varies according to the social organization of the ethnic communities. We assume that there would be significant differences between the women’s and men’s exposure to different types of MV among the various ethnic societies.

## Methods

### Study design

We used a dataset of an intervention study entitled “Community-based prevention of domestic violence among Bengali, Garo, and Santal ethnic communities.” The study followed a cluster-randomized control trial (C-RCT) with assessments at baseline and follow-up 12 months after the intervention. The intervention incorporates capacity-building workshops, community education, and support-group formations. We conducted the baseline survey in 24 villages from February to May 2019. The intervention was implemented in 12 villages between November 2019 and February 2020. The current study is based on the pre-intervention survey. The main study examined the effectiveness of the community-based domestic violence prevention intervention. Gender-disaggregated exposure to MV was used as one of the outcome measures. Although the main study is longitudinal, this study follows a cross-sectional design. However, there is no possibility that the intervention will influence the result of this study.

### Study sites

The current study included ethnic minority *Garo* and *Santal* and mainstream *Bengali* communities. The fieldwork was conducted in 24 purposively selected villages (eight *Santal*, eight *Bengali*, and eight *Garo* villages) located in the different regions of Bangladesh. The *Bengali* villages were located in two sub-districts in northwest Bangladesh. Both the sub-districts were situated 15–20 km northeast of Rajshahi city. The *Santal* villages were selected from another northwest sub-district, where the indigenous people settled about 300 years ago. The villages were 20–30 km northwest of the Rajshahi. On the other hand, the *Garo* villages were selected from a northeast sub-district where they lived for more than 500 years. These villages were nearly 250km northeast of Rajshahi. There are about 68,000 villages in Bangladesh; thus, it was beyond our time and budget to embrace a statistically representative number of villages. However, we selected our participants from the villages using a random sampling method.

### Study participants

The study included currently married men and women aged 16–60-years-old, married for at least one year. There were a few girls below 16 years of age, but none were married for a year or more. Because of the lack of appropriate facilities, we also excluded individuals with mental disorders and physical disabilities (e.g., deaf/mute). The sample size was determined for the baseline survey. Considering the proportion of sexual abuse, based on our pilot study in an ethnic village (*p* = 0.167), we estimated the minimum required sample size at 1,854 using a formula (n = z^2^_α/2_ p(1-p)/E^2^, where p = proportion; α = 0.05; therefore, z _α/2_ = 1.96; E = p/10) [30]. To avoid non-consent/drop out, we created a sample pool with 10% over samples. Altogether, we approached 1,968 persons. We used a cluster sampling procedure to select our study participants.

At first, we identified the study villages. After selecting a village, we collected an updated list of their households. Each of the villages consists of roughly 100−300 households. From them, we randomly selected the first household in the village. In order to form a cluster, we then included 81 additional households close to the first household. These households were the most physically nearest households from the household we selected at first. To do so, we conducted a quick household mapping in the area. After that, we randomly assigned half of the households for male respondents and the other half for female respondents. As a final point, we approached one respondent (a man or a woman) from a household for face-to-face interviews. If a household had more than one eligible respondent, we randomly selected one of them. In total, 1,929 respondents (961 men and 968 women) completed the questionnaire. The response rate was 98.02%. Reasons for non-participation were mostly related to the respondents’ lack of time.

### Dependent variable

#### Exposure to marital violence

A revised version of the NorVold Abuse Questionnaire (NorAQ) was used to assess lifetime experiences of MV [31]. We used three variables: (a) experiences of emotional abuse, (b) experiences of physical abuse, and (c) experiences of sexual abuse. All of the variables had two categories: 0 = none and 1 = yes. The ‘yes’ category was further classified: 1 = mild abuse and 2 = severe abuse. The scale incorporated 26 items on different types of victimization (see S1 File). We considered how the respondents experienced different types of abuse (e.g., emotional, physical, and sexual) from their spouses. The notable feature of NorAQ is that it specifies the severity of abuse, namely mild abuse and severe abuse [31]. As explained by Katarina Swahnberg [31], the content of the items used in the NorAQ ranges from mild to severe lifetime abuse, allowing for a rough categorization of the severity of any abusive act. Although the original scale suggested three categories of the severity of abuse, such as mild, moderate, and severe [31], we merged mild and moderate categories as ‘mild abuse’ in the current study. Thus, we had two categories: mild abuse and severe abuse (see S1 File).

Regarding the nature of abuse, we considered some abusive acts as ‘mild abuse,’ while others as ‘severe abuse’ [31]. Questions on mild emotional abuse included humiliating behaviors, e.g., ‘has your spouse displayed anger or hatred at you?’ For severe emotional abuse, questions were posed on threatening and intimidating behaviors, such as ‘has he/she threatened to kill or injure you seriously.’ On the other hand, questions on mild physical abuse included: ‘has he/she twisted your arm/hair or slapped you,’ while the example of severe physical abuse was: ‘has he/she kicked or beaten you with a stick or something else.’ An item of *mild sexual MV* was ‘has he/she touched your sexual organs (breast/vagina/penis) against your will,’ while *severe sexual MV* included: ‘has he/she compelled you to participate in sex when you were not interested.’

Each of the questions was rated with the responses: ‘0 = no,’ or ‘1 = yes.’ Finally, we constructed the variables by considering relevant severity scores, e.g., 0 = none (non-exposed), 1 = mild (exposed to mild abuse only), and 2 = severe (exposed to any severe abuse–including those who experienced both severe and mild cases of abuse). We also constructed two more variables: any lifetime MV (experienced at least one type of MV) and poly-victimization (had all three types of MV). The NorAQ was developed in a western context [31], while we validated the scale in a pilot study [32]. In this study, the NorAQ showed high consistency: α = 0.86 (emotional sub-scale α = 0.70, physical sub-scale α = 0.63, and sexual sub-scale α = 0.80). We also conducted a confirmatory factor analysis of the items, which supported the second-order factorial solution.

### Explanatory variables

The main explanatory variables were ethnicity and gender. We also used age, education, occupation, monthly income, and family structure. Ethnicity had three categories: *Garo*, *Santal*, and *Bengali*. According to the social organization, these communities were further classified as matrilineal and patrilineal. Gender had two categories: men and women. Age was classified: 16–25 years, 26–35 years, 36–45 years, and 46–60 years. The minimum age of the study participants was 16 years (*F* = 16 years, *M* = 19 years), while the maximum age was 60 years (*F* = 54 years, *M* = 60 years). In the sample, only 15 (out of 969) women were ≤19-years-old, and only 15 (out of 960) men were ≤25-years-old. Therefore, it was not deemed feasible to have a separate category for the adolescent respondents. According to the typical categorization of educational attainment in Bangladesh, it had four levels: No schooling, Primary (1–5 years of schooling), Secondary (6–10 years of schooling), and Higher (11+ years–passed the SSC examination or above). The occupations were categorized as unemployed (having no formal source of income, although this may include homemaking jobs), agric farming (earning from agriculture), day laborers (earning daily by selling labor in farming, transport, or other industries), and jobs and others (earning from jobs, business, or others). Monthly income was classified into three categories concerning the poverty line income in the Bangladesh context: 1 = no cash income/not earning an income, 2 = earning less than US$30, and 3 = earning US$30 or above. The family structure had two categories: Nuclear (constituted of husband and wife, and/or unmarried children) and extended (having husband and wife, married adult children, and/or in-laws).

### Data collection

We used a structured questionnaire. At first, we conducted a pilot study with 331 samples to validate the study measures. After that, we further pre-tested the retained items before the baseline survey. The questionnaire was administered in face-to-face interviews. This interview allowed the interviewer to ask questions in person, ensuring a better quality response [33]. The study participants were contacted in person in their homes. Four graduates in social work (two males and two females) were employed to collect the data. Male interviewers interviewed the male respondents, while female interviewers interviewed the female respondents, respectively.

Due to the sensitive nature of the data, interviewers were trained on ethical, safety, and technical issues related to the data collection. We emphasized the importance of establishing rapport with the respondents. We started with less-sensitive questions, which allowed the respondents to adapt more easily to the sensitive issues. We also compensated the participants for their valuable time. Although there is some debate about compensating respondents, we believed it was the right thing to do since we took a fair amount of the respondents’ time. We observed that many study participants, particularly from the ethnic minority communities, were engaged in day-laboring the whole day, challenging them to devote any time to other purposes. Hence, we felt an ethical responsibility to offer them compensation. These factors (establishing rapport, orderly questioning, and compensation) encouraged them to participate in the study.

### Data analysis

The purpose of the data analysis was to estimate the ethnic- and gender-specific features of MV. We computed descriptive statistics for all the study variables. Cross-tabulations were used to provide the ethnic- and gender-specific distributions for the socio-demographic profiles of the respondents. We also prepared multivariate tables to depict the prevalence of different types of MV. Bivariate cross-tabulations were conducted to determine the relationships between gender/ethnicity and MV. Finally, we employed multivariate logistic regression in order to assess the gender and ethnic differences in the different types of MV [34]. Binary dependent variables, independent observations, no multicollinearity among the variables used in the model, and a large sample size met the assumptions of logistic regression. Coefficients of logistic regression with log link function produced Odds Ratio (OR), which facilitated the interpretation of the regression outcomes [34]. The data analysis was conducted using SPSS 23.0 software [35].

### Ethical procedures

We conducted the study following the WHO guidelines for researching violence against women [36]. The study protocol was approved by the Ethics Review Committee at the Faculty of Social Sciences, the University of Rajshahi, Bangladesh (Approval number: 107(3)/ FSS/RU -EC/ 2020). Respondents were informed about the protocol. Informed consent was obtained verbally. We read out the consent statement before conducting the interview. Respondents were informed that they might find some questions uncomfortable. We reminded them repeatedly that their participation was voluntary and that she/he had no obligation to complete the interview and could drop out at any time. The interviewers were given training in the basic caring skills to help survivors of MV. Only one respondent (either male or female) from a household was selected, avoiding any possible discomfort among the family. Anonymity and confidentiality were maintained. We also informed the participants about our future intervention. We provided them our contact details so that anyone could contact us for help or if they needed any information. We were contacted by some of the abused women later, wherein the team supported them. We referred the women to the support centers/organizations available in the area (see S2 File).

### Public involvement

Community representatives/leaders participated in the design and implementation of our intervention in order to prevent marital violence in their area. However, it was not appropriate or possible to involve them in the design of the baseline survey or to conduct it; nonetheless, they later participated in the dissemination of our research findings in their communities.

## Results

### Sample characteristics

Table 2 presents the socio-demographic profile of the respondents, as well as their ethnic and gender distributions. Of the sample (*N* = 1929), 33.2% (*n* = 640) were from *Garo*, 33.2% (*n* = 640) from *Santal*, and 33.6% (*n* = 649) from the *Bengali* ethnic communities. Almost half (50.2%) of the respondents were women; the majority (38.7%) of them belonged to a younger (26–35 years) age group; and most (46.9%) had been married between 11 and 20 years. By education, 42.5% had attained primary education, 29.8% had secondary education, and only 20.0% had a higher level of education. A majority of them (41.3%) were day laborers; and 48.3% earned a monthly income of US$30 or above. The table also shows that there were both ethnic and gender differences in the respondents’ socioeconomic status (see Table 2).

**Table 2:** Demographic profile and socioeconomic status of the study participants by gender and ethnicity.

	Overall				Men				Women			
	Total	Garo	Santal	Bengali	Total	Garo	Santal	Bengali	Total	Garo	Santal	Bengali
	N = 1929 (%)	N = 640 (%)	N = 640 (%)	N = 649 (%)	n = 960 (%)	n = 318 (%)	*n* = 319 (%)	n = 323 (%)	n = 969 (%)	n = 322 (%)	n = 321 (%)	n = 326 (%)
**Age in years**												
16–25	301 (15.6)	71 (11.1)	127 (19.8)	103 (15.9)	34 (3.5)	8 (2.5)	17 (5.3)	17 (5.3)	267 (27.6)	63 (19.6)	110 (34.3)	110 (34.3)
26–35	746 (38.7)	220 (34.4)	273 (42.7)	253 (39.0)	300 (31.3)	82 (25.8)	117 (36.7)	117 (36.7)	446 (46.0)	138 (42.8)	156 (48.6)	156 (48.6)
36–45	711 (36.8)	230 (35.9)	212 (33.1)	269 (41.4)	487 (50.7)	140 (44.0)	157 (49.2)	157 (49.2)	224 (23.1)	90 (28.0)	55 (17.1)	55 (17.1)
46–60	171 (8.9)	119 (18.6)	28 (4.4)	24 (3.7)	139 (14.5)	88 (27.7)	28 (8.8)	28 (8.8)	32 (3.3)	31 (9.6)	0 (0.0)	0 (0.0)
**Years married**												
1–10	679 (35.2)	225 (35.2)	260 (40.6)	194 (29.9)	267 (27.8)	87 (27.4)	98 (30.7)	82 (25.4)	412 (42.5)	138 (42.8)	162 (50.5)	112 (34.4)
11–20	904 (46.9)	244 (38.1)	319 (49.9)	341 (52.5)	531 (55.3)	144 (45.3)	187 (58.6)	200 (61.9)	373 (38.5)	100 (31.1)	132 (41.1)	141 (43.2)
21–38	346 (17.9)	171 (26.7)	61 (9.5)	114 (17.6)	162 (16.9)	87 (27.3)	34 (10.7)	41 (12.7)	184 (19.0)	84 (26.1)	27 (8.4)	73 (22.4)
**Education**												
No schooling	149 (7.7)*	28 (4.4)	76 (11.9)	45 (6.9)	51 (5.3)*	13 (4.1)	27 (8.5)	11 (3.4)	98 (10.1)*	15 (4.7)	49 (15.3)	34 (10.4)
Primary	820 (42.5)	278 (43.4)	304 (47.5)	238 (36.7)	457 (47.6)	161 (50.6)	171 (53.6)	125 (38.7)	363 (37.5)	117 (36.3)	133 (41.4)	113 (34.7)
Secondary	575 (29.8)	188 (29.4)	166 (25.9)	221 (34.1)	228 (30.0)	94 (29.6)	90 (28.2)	104 (32.2)	287 (29.6)	94 (29.2)	76 (23.7)	117 (35.9)
Higher	385 (20.0)	146 (22.8)	94 (14.7)	145 (22.3)	164 (17.1)	50 (15.7)	31 (9.7)	83 (25.7)	221 (22.8)	96 (29.8)	63 (19.6)	62 (19.0)
**Main occupation**												
Home-making	636 (33.0)*	167 (26.1)	153 (23.9)	316 (48.7)	3 (0.3)*	3 (0.9)	0 (0.0)	0 (0.0)	633 (65.3)*	164 (50.9)	153 (47.7)	316 (97.0)
Day laborers	797 (41.3)	265 (41.4)	435 (68.0)	97 (14.9)	537 (55.9)	164 (51.6)	278 (87.2)	95 (29.4)	260 (26.8)	101 (31.4)	157 (48.9)	2 (0.6)
Agric farming	329 (17.0)	154 (24.1)	29 (4.5)	146 (22.5)	281 (29.3)	116 (36.5)	25 (7.8)	140 (43.3)	48 (5.0)	38 (11.8)	4 (1.2)	6 (1.8)
Job and others	167 (8.7)	54 (8.4)	23 (3.6)	90 (13.9)	139 (14.5)	35 (11.0)	16 (5.0)	88 (27.3)	28 (2.9)	19 (5.9)	7 (2.2)	2 (0.6)
**Monthly income**												
No income	423 (21.9)*	101 (15.8)	119 (18.6)	203 (31.3)	2 (0.2)*	2 (0.6)	0 (0.0)	0 (0.0)	421 (43.4)*	99 (30.7)	119 (37.1)	203 (62.2)
Less than $30	575 (29.8)	134 (20.9)	289 (45.2)	152 (23.4)	177 (18.4)	15 (4.7)	122 (38.2)	40 (12.4)	398 (41.1)	119 (37.0)	167 (52.0)	112 (34.4)
$30 and above	931 (48.3)	405 (63.3)	232 (36.2)	294 (45.3)	781 (81.4)	301 (94.7)	197 (61.8)	283 (87.6)	150 (15.5)	104 (32.3)	35 (10.9)	11 (3.4)
**Family structure**												
Nuclear	1411 (73.1)*	394 (61.6)	511 (79.8)	506 (78.0)	738 (76.9)*	206 (64.8)	274 (85.9)	258 (79.9)	673 (69.5)*	188 (58.4)	237 (73.8)	248 (76.1)
Extended	518 (26.9)	246 (38.4)	129 (20.2)	143 (22.0)	222 (23.1)	112 (35.2)	45 (14.1)	65 (20.1)	296 (30.5)	134 (41.6)	84 (26.2)	78 (23.9)

Note: Results of χ^2^ tests and residual analysis by gender and ethnicity are presented in the S1 Table.

*Significance at <0.001 level.

The ethnic minority *Garo* was matrilineal by social organization, whereas both the *Bengali* and *Santal* communities were patrilineal. Of the sample, it appeared that 33.2% (*n* = 640) were matrilineal, while 66.8% (*n* = 1289) were patrilineal (see Table 4). Data also revealed that the socioeconomic status of the women was relatively high among the ethnic communities than the mainstream *Bengali* community (see Table 2). The rate of higher education among women was better in the *Garo* (29.8%) than in the *Santal (*19.6%) and *Bengali* (19.0%) societies. While 97.0% of the women in the *Bengali* community confined themselves to household chores, most of the women in both the *Garo* and *Santal* communities were engaged in different jobs outside the home boundary. Several women (32.3%) among the matrilineal *Garo* community also earned US$30 or more per month, while the rate was quite low among the patrilineal communities (*Santal* = 10.9%, *Bengali =* 3.4%). On the other hand, data revealed that most of the women (62.2%) in the patrilineal *Bengali* community had no cash income (see Table 2).

### Experience of marital violence by gender

Data revealed that the prevalence of different types of MV was quite high in the sample: 52.8% of them experienced emotional abuse (mild = 22.3%, severe = 30.5%), 32.3% experienced physical abuse (mild = 14.6%, severe = 17.7%), and 36.0% experienced sexual abuse (mild = 3.3%, severe = 32.7%) from their spouse (see Table 3). Data also showed that 53.6% of them were exposed to any type of lifetime MV, while 25.4% of the respondents experienced poly-victimization (see Table 3).

**Table 3:** The lifetime prevalence of different types of marital victimization among the respondents by gender and ethnicity.

	Overall				Men				Women			
	Total	Garo	Santal	Bengali	Total	Garo	Santal	Bengali	Total	Garo	Santal	Bengali
	N = 1929 (%)	N = 640 (%)	N = 640 (%)	N = 649 (%)	n = 960 (%)	n = 318 (%)	*n* = 319 (%)	n = 323 (%)	n = 969 (%)	n = 322 (%)	n = 321 (%)	n = 326 (%)
**Emotional violence**												
No	910 (47.2)	297 (46.4)	307 (48.0)	306 (47.2)	867 (90.3)	267 (84.0)	305 (95.6)	295 (91.3)	43 (4.4)	30 (9.3)	2 (0.6)	11 (3.4)
Yes	1019 (52.8)	343 (53.6)	333 (52.0)	343 (52.8)	93 (9.7)	51 (16.0)	14 (4.4)	28 (8.7)	926 (95.6)	292 (90.7)	319 (99.4)	315 (96.6)
Mild	431 (22.3)	182 (28.4)	136 (21.2)	113 (17.4)	61 (6.4)	32 (10.0)	12 (3.8)	17 (5.3)	370 (38.2)	150 (46.6)	124 (38.6)	96 (29.4)
Severe	588 (30.5)	161 (25.2)	197 (30.8)	230 (35.4)	32 (3.3)	19 (6.0)	2 (0.6)	11 (3.4)	556 (57.4)	142 (44.1)	195 (60.8)	219 (67.2)
**Physical violence**												
No	1307 (67.7)	463 (72.3)	423 (66.1)	421 (64.9)	953 (99.3)	313 (98.5)	318 (99.7)	322 (99.7)	354 (36.5)	150 (46.6)	105 (32.7)	99 (30.4)
Yes	622 (32.3)	177 (27.7)	217 (33.9)	228 (35.1)	7 (0.7)	5 (1.5)	1 (0.3)	1 (0.3)	615 (63.5)	172 (53.4)	216 (67.3)	227 (69.6)
Mild	281 (14.6)	70 (11.0)	99 (15.5)	112 (17.2)	5 (0.5)	3 (0.9)	1 (0.3)	1 (0.3)	276 (28.5)	67 (20.8)	98 (30.5)	111 (34.0)
Severe	341 (17.7)	107 (16.7)	118 (18.4)	116 (17.9)	2 (0.2)	2 (0.6)	0 (0.0)	0 (0.0)	339 (35.0)	105 (32.6)	118 (36.8)	116 (35.6)
**Sexual violence**												
No	1236 (64.0)	434 (67.8)	411 (64.2)	391 (60.2)	959 (99.9)	318 (100.0)	319 (100.0)	322 (99.7)	277 (28.6)	116 (36.0)	92 (28.7)	69 (21.2)
Yes	693 (36.0)	206 (32.2)	229 (35.8)	258 (39.8)	1 (0.1)	0 (0.0)	0 (0.0)	1 (0.3)	692 (71.4)	206 (64.0)	229 (71.3)	257 (78.8)
Mild	63 (3.3)	22 (3.5)	20 (3.1)	21 (3.3)	1 (0.1)	0 (0.0)	0 (0.0)	1 (0.3)	62 (6.4)	22 (6.8)	20 (6.2)	20 (6.1)
Severe	630 (32.7)	184 (28.7)	209 (32.7)	237 (36.5)	0 (0.0)	0 (0.0)	0 (0.0)	0 (0.0)	630 (65.0)	184 (57.2)	209 (65.1)	237 (72.7)
**Overall violence**												
**Any type (yes)**	1033 (53.6)	351 (54.8)	333 (52.0)	349 (53.8)	95 (9.9)	52 (16.4)	14 (4.4)	29 (9.0)	938 (96.8)	299 (92.9)	319 (99.4)	320 (98.2)
**Poly abuse (yes)**	490 (25.4)	125 (19.5)	173 (27.0)	192 (29.6)	0 (0.0)	0 (0.0)	0 (0.0)	0 (0.0)	490 (50.6)	125 (38.8)	173 (53.9)	192 (58.9)

Note: Results of χ^2^ tests, residual analysis, and the 95% CI of the ratios of MV by gender and ethnicity are presented in the S2 Table.

We observed a big gender difference in the exposure to different types of MV. Data showed that women were the primary subjects of MV in almost all cases. Only a few men experienced mild emotional abuse from their spouses. It appeared that men were not subject to physical and sexual MV from their wives. The prevalence of emotional abuse was very high among women (95.6%, including severe abuse = 57.4%) compared to men (9.7%, including severe abuse = 3.3%), χ^2^ = 1434.62, *p*<0.001. The occurrence of physical victimization among women was also widespread (63.5%, including severe abuse = 35.0%), while it was very negligible among men (0.7%, including severe abuse = 0.2%), χ^2^ = 868.90, *p*<0.001. Only the women were exposed to sexual MV (71.4%, including severe abuse = 65.0%), whereas almost no men experienced it (0.1%, no severe abuse reported), χ^2^ = 1065.36, *p*<0.001. The study also revealed that the prevalence of any type of lifetime MV against women was 96.8%, while it was only 9.9% among men, χ^2^ = 1343.07, *p*<0.001. We also observed that no men experienced poly victimization, whereas 50.6% of the women experienced it (see Table 3). The results of the post hoc/residual analysis are presented in the S2 Table.

### Experience of marital violence by ethnicity

We observed ethnic differences in the experience of MV. Although the prevalence of any MV was not higher in the mainstream *Bengali* community (53.8%) compared to the ethnic *Garo* (54.8%) and *Santal* (52.0%) communities (*χ*^*2*^ = 1.04, *p* = 0.595), the study revealed that the prevalence of poly-victimization among the *Bengali* community (29.6%) was higher than the *Santal* (27.0%) and the *Garo* (19.5%) ethnic communities (*χ*^*2*^ = 18.53, *p*<0.001) (see Table 3).

It appeared that the *Garo* respondents experienced less severe-type emotional MV (25.2%) than the *Santal* (30.8%) and *Bengali* communities (35.4%), χ^2^ = 29.418, p<0.001. The respondents from the *Garo* community also experienced less physical MV (27.7%) than the *Santal* (33.9%) and the *Bengali* communities (35.1%), χ^2^ = 12.97, *p* = 0.011. We also observed that the prevalence of sexual MV was low among the *Garo* community (32.2%) compared to the *Santal* (35.8%) and the *Bengali* communities (39.8%), χ^2^ = 8.93, *p* = 0.063 (see Table 3).

Furthermore, we found that all types of MV against women were widespread among ethnic communities. Although the *Garo* women experienced a relatively lower rate of emotional MV, it was still very high among the three ethnic communities (*Garo* = 90.7%, *Santal* = 99.4%, *Bengali* = 96.6%), χ^2^ = 57.100, *p*<0.001. In contrast, the rate of emotional MV was higher among the *Garo* men (16.0%, including severe abuse = 6.0%) than both the *Santal* (4.4%, including severe abuse = 0.6%) and the *Bengali* men (8.7%, including severe = 3.4%), χ^2^ = 26.98, *p*<0.001.

The study also showed that almost no men from any communities were subject to severe physical violence (*Garo* = 0.6%, *Santal* = 0.0%, and *Bengali* = 0.0%). Only a negligible number of men experienced physical MV from their wives (*Garo* = 1.5%, *Santal* = 0.3%, *Bengali* = 0.3%), χ^2^ = 5.70, *p* = 0.223. Yet, most of the women experienced physical MV in all of the ethnicities, wherein the *Garo* women were exposed to a relatively lower rate of physical abuses (53.4%, including severe abuse = 32.6%) than the *Santal* (67.3%, including severe abuse = 36.8%) and *Bengali* women (69.6%, including severe abuse = 35.6%), χ^2^ = 25.10, *p*<0.001 (see Table 3).

The data also revealed that the men did not experience sexual coercion from their wives. Only one man (0.3%) from the *Bengali* community reported mild sexual abuse from his wife. On the other hand, women from all ethnicities were largely exposed to sexual MV by their husbands (*Garo* = 64.0%, *Santal* = 71.3%, *Bengali* = 78.8%). Rather than mild sexual abuse (6.4%), the study showed that women were more exposed to severe sexual MV (65.0%). Yet, the *Garo* women experienced a lower rate of severe sexual abuse (57.2%) than the women from the *Santal* (65.1%) and the *Bengali* (72.7%) ethnic communities, χ^2^ = 18.69, *p* = 0.001(see Table 3).

The study further revealed that none of the husbands from any of the ethnic communities experienced poly-victimization. Most of the abused men experienced only one type of MV, where the *Garo* men reported more abuse (*Garo* = 16.4%, *Santal* = 4.4%, *Bengali* = 9.0%), χ^2^ = 26.02, *p*<0.001. In contrast, a majority of the women experienced poly-victimization, where the *Garo* women had a lower rate (38.8%) than the *Santal* (53.9%) and *Bengali* women (58.9%), χ^2^ = 28.24, *p*<0.001 (see Table 3). The 95% CI of the ratios of MV by gender and ethnicity are presented in the S2 Table.

### Experience of marital violence by social organization

Regarding social organization, the study included two types of ethnic communities: matrilineal and patrilineal. The *Garo* is a matrilineal society (sometimes referred to as a matriarchal society), whereas both the *Bengali* and *Santal* communities are examples of a patrilineal (patriarchal) society (see Table 1). We observed that the respondents in the matrilineal *Garo* society experienced less MV than the patrilineal *Bengali* and *Santal* societies. It appeared that the respondents from the matrilineal society experienced a relatively lower rate of severe emotional abuse within the marriage (25.2%) compared to the patrilineal society (33.1%), *χ*^*2*^ = 24.95, *p*<0.001. The rates of physical abuse (27.7%) appeared to be low among the matrilineal society than the patrilineal society (34.5%), *χ*^*2*^ = 12.14, *p* = 0.005 (see Table 4). The respondents from the matrilineal society experienced less sexual abuse (32.2%) than the patrilineal society (37.8%), *χ*^*2*^ = 6.66, *p* = 0.015 (see Table 4).

**Table 4:** The lifetime prevalence of different types of marital victimization by gender and social organization.

	Overall				Men				Women			
	Total	Matrilineal	Patrilineal	χ^2^	Total	Matrilineal	Patrilineal	χ^2^	Total	Matrilineal	Patrilineal	χ^2^
	N = 1929 (%)	n = 640 (%)	n = 1289 (%)		n = 960 (%)	n = 318 (%)	n = 642 (%)		n = 969 (%)	n = 322 (%)	*n* = 647 (%)	
**Emotional violence**				24.95***				22.37***				49.61***
No	910 (47.2)	297 (46.4)	613 (47.6)		867 (90.3)	267 (84.0)	600 (93.5)		43 (4.4)	30 (9.3)	13 (2.0)	
Yes	1019 (52.8)	343 (53.6)	676 (52.4)		93 (9.7)	51 (16.0)	42 (6.5)		926 (95.6)	292 (90.7)	634 (98.0)	
Mild	431 (22.3)	182 (28.4)	249 (19.3)		61 (6.4)	32 (10.0)	29 (4.5)		370 (38.2)	150 (46.6)	220 (34.0)	
Severe	588 (30.5)	161 (25.2)	427 (33.1)		32 (3.3)	19 (6.0)	13 (2.0)		556 (57.4)	142 (44.1)	414 (64.0)	
**Physical violence**				12.14**				5.70*				24.08***
No	1307 (67.8)	463 (72.3)	844 (65.5)		953 (99.3)	313 (98.5)	640 (99.7)		354 (36.5)	150 (46.6)	204 (31.5)	
Yes	622 (32.2)	177 (27.7)	445 (34.5)		7 (0.7)	5 (1.5)	2 (0.3)		615 (63.5)	172 (53.4)	443 (68.5)	
Mild	281 (14.6)	70 (11.0)	211 (16.4)		5 (0.5)	3 (0.9)	2 (0.3)		276 (28.5)	67 (20.8)	209 (32.3)	
Severe	341 (17.6)	107 (16.7)	234 (18.1)		2 (0.2)	2 (0.6)	0 (0.0)		339 (35.0)	105 (32.6)	234 (36.2)	
**Sexual violence**				6.66*				0.49				14.07***
No	1236 (64.1)	434 (67.8)	802 (62.2)		959 (99.9)	318 (100.0)	641 (99.8)		277 (28.6)	116 (36.0)	161 (24.9)	
Yes	693 (35.9)	206 (32.2)	487 (37.8)		1 (0.1)	0 (0.0)	1 (0.2)		692 (71.4)	206 (64.0)	486 (75.1)	
Mild	63 (3.3)	22 (3.4)	41 (3.2)		1 (0.1)	0 (0.0)	1 (0.2)		62 (6.4)	22 (6.8)	40 (6.2)	
Severe	630 (32.6)	184 (28.8)	446 (34.6)		0 (0.0)	0 (0.0)	0 (0.0)		630 (65.0)	184 (57.2)	446 (68.9)	
**Overall violence**												
**Any type (yes)**	1033 (53.6)	351 (54.8)	682 (52.9)	0.64	95 (9.9)	52 (16.4)	43 (6.7)	22.23***	938 (96.8)	299 (92.9)	639 (98.8)	24.21***
**Poly victimization (yes)**	490 (25.4)	125 (19.5)	365 (28.3)	17.42***	0 (0.0)	0 (0.0)	0 (0.0)	0.0	490 (50.6)	125 (38.8)	365 (56.4)	26.62***

Note: The ethnic minority *Garo* is matrilineal, while the mainstream *Bengali* and ethnic minority *Santal* are patrilineal.

*significance at 0.05 level

**significance at 0.01 level

***significance at <0.001 level

The study further revealed that married men in the matrilineal society experienced a higher degree of emotional (16%) and physical (1.5%) abuse from their wives compared to men in the patrilineal society (emotional = 6.5%, physical = 0.3%). On the other hand, men were not subject to sexual MV in either the matrilineal or the patrilineal society (see Table 4).

Data also showed that women from the matrilineal society were less exposed to all types of MV than the patrilineal society. The rate of emotional MV against women appeared to be lower in the matrilineal society (overall = 90.7%, severe abuse = 44.1%) than the patrilineal society (overall = 98.0%, severe abuse = 64.0%), *χ*^*2*^ = 49.61, *p*<0.001. The matrilineal society also had lower rates of physical MV against women (overall = 53.4%, severe abuse = 32.6%) than the patrilineal society (overall = 68.5%, severe abuse = 36.2%), *χ*^*2*^ = 24.08, *p*<0.001. Data also showed that women experienced less sexual MV in the matrilineal society (overall = 64.0%, severe/marital rape = 57.2%) than the patrilineal society (overall = 75.1%, severe/marital rape = 68.9%), *χ*^*2*^ = 14.07, *p*<0.001 (see Table 4).

The study further revealed that no men experienced poly-victimization from their spouse in the matrilineal or the patrilineal society. Most of the men experienced only one type of MV, where men from the matrilineal society reported more abuse than the patrilineal society (matrilineal = 16.4%, patrilineal = 6.7%), *χ*^*2*^ = 22.23, *p*<0.001. In contrast, a majority of the women experienced poly-victimization (50.6%). However, women in the matrilineal society experienced less poly-victimization (38.8%) than the patrilineal society (56.4%), *χ*^*2*^ = 26.62, *p*<0.001 (see Table 4).

### Multivariate models

We also conducted multivariate logistic regressions to explore the adjusted effects of gender and ethnicity on different types of MV. We used robust estimation to minimize the influence of extreme observations (e.g., only a few men experienced MV). This resulted in quite stable estimates without losing the data character [37]. Table 5 represents four models for estimating: (a) experience of emotional MV, (b) experience of physical MV, (c) experience of sexual MV, and (e) experience of any type of MV. It also shows the effects of adjusting factors, e.g., age, education, occupation, income, and family structure, on the MV (see Table 5). To avoid multicollinearity issues, we excluded ‘social organization’ from the final models. The social organization appeared to be highly correlated with ethnicity. We kept ethnicity in the model and set *Garo* as the reference category. This also enabled us to assess the differences in the occurrence of MV between the matrilineal and patrilineal societies to a greater extent.

**Table 5:** Multivariate logistic regression models for estimating ethnic and gender differences in different types of MV.

	Emotional			Physical			Sexual			Any Abuse		
	OR	95% CI	P-value	OR	95% CI	P-value	OR	95% CI	P- value	OR	95% CI	P-value
**Gender**												
Women	184.44	93.65−363.24	<0.001	449.23	181.59−1111.35	<0.001	2789.71	381.36−20407.08	<0.001	294.95	133.87−649.84	<0.001
Men (ref)	1			1			1			1		
**Ethnicity**												
Bengali	0.87	0.49−1.53	0.630	1.90	1.27−2.85	0.002	2.04	1.34−3.10	0.001	0.87	0.49−1.55	0.641
Santal	0.76	0.44−1.30	0.314	1.38	0.93−2.05	0.111	1.19	0.80−1.77	0.382	0.61	0.34−1.08	0.090
Garo (ref)	1			1			1			1		.
**Age in years**												
46–60	0.96	0.27−3.43	0.943	1.51	0.54−4.28	0.434	0.95	0.33−2.72	0.921	1.00	0.26−3.87	0.998
36–45	0.90	0.36−2.25	0.818	1.59	0.83−3.02	0.159	1.35	0.68−2.68	0.385	0.83	0.31−2.28	0.724
26–35	0.97	0.50−1.87	0.920	1.23	0.82−1.85	0.320	1.17	0.75−1.83	0.487	0.98	0.46−2.06	0.952
16–25 (ref)	1			1			1			1		
**Years married**												
21–38	1.94	0.81−4.65	0.140	1.09	0.56−2.11	0.798	0.73	0.36−1.49	0.393	1.58	0.63−3.96	0.324
11–20	1.22	0.71−2.11	0.475	1.34	0.89−2.01	0.165	0.97	0.63−1.51	0.894	1.08	0.63−1.88	0.772
1–10 (ref)	1			1			1			1		
**Education**												
None	0.83	0.41−1.68	0.604	2.22	1.25−3.94	0.006	1.49	0.84−2.65	0.169	0.80	0.37−1.72	0.571
Primary	0.99	0.54−1.81	0.967	3.92	2.64−5.81	<0.001	1.88	1.27−2.77	0.002	1.10	0.59−2.08	0.759
Secondary	1.15	0.64−2.07	0.645	2.59	1.77−3.78	<0.001	2.04	1.36−3.04	0.001	1.21	0.66−2.23	0.541
Higher (ref)	1						1			1		
**Main occupation**												
Job/others	4.09	1.51−11.11	0.006	1.78	0.64−4.99	0.270	0.50	0.22−1.18	0.114	4.14	1.37−12.51	0.012
Agric farming	2.05	0.91−4.62	0.082	1.67	1.03−2.71	0.039	1.31	0.80−2.14	0.275	2.18	0.85−5.61	0.106
Day laborers	1.82	0.71−4.68	0.217	1.26	0.61−2.62	0.528	0.69	0.33−1.47	0.342	1.86	0.63−5.47	0.259
Home-making (ref)	1			1			1			1		
**Monthly income**												
No income	3.85	1.56−9.51	0.003	1.43	0.80−2.57	0.230	1.08	0.60−1.96	0.799	2.79	1.00−7.75	0.048
Less than $30	1.98	1.24−3.16	0.004	1.88	1.19−2.98	0.007	1.02	0.63−1.64	0.952	1.89	1.14−3.11	0.013
$30/above (ref)	1			1			1			1		
**Family structure**												
Nuclear	0.68	0.42−1.10	0.121	1.62	1.18−2.21	0.003	0.81	0.58−1.14	0.228	0.64	0.39−1.05	0.075
Extended (ref)	1			1			1			1		
**Model summary**												
Likelihood Ratio	*χ*^*2*^ = 1729.43, *df* = 17, *p*<0.001			*χ*^*2*^ = 1214.06, *df* = 17, *p*<0.001			*χ*^*2*^ = 1390.66, *df* = 17, *p*<0.001			*χ*^*2*^ = 1793.66, *df* = 17, *p*<0.001		
Log Likelihood	-334.101			-364.924			-311.934			-308.345		

#### Gender differences in MV

Data showed a significant gender difference in the exposure to different types of MV. After holding other variables constant, it appeared that the ORs for emotional, physical, sexual, and any type of MV were, respectively, 184.44 (95% CI = 93.65−363.24, p<0.001), 449.23 (95% CI = 181.59−1111.35, p<0.001), 2789.71 (95% CI = 381.36−20407.08, p<0.001), and 294.95 (95% CI = 133.87−649.84, p<0.001) for the women compared to the men (see Table 5).

#### Ethnic differences in MV

The study also revealed significant ethnic differences in the exposure to physical and sexual MV. Data showed that the patriarchal Bengali community experienced a higher degree of physical (OR = 1.90, 95% CI = 1.27−2.85, p = 0.002) and sexual abuse (OR = 2.04, 95% CI = 1.34−3.10, p<0.001) than the matrilineal *Garo* community. However, we did not find any significant differences between the matrilineal *Garo* and the patrilineal *Santal* communities in the exposure to physical and sexual MV. We also did not observe any significant variations in the emotional abuse and any type of marital victimization among the communities (see Table 5).

#### Role of controlled variables

In addition to gender and ethnicity, the study further revealed that education, income, and family structure had significant influences on the exposure to MV in the sample. Data showed that the respondents with no education/schooling (OR = 2.22, 95% CI = 1.25−3.94, p = 0.006), primary education (OR = 3.92, 95% CI = 2.64−5.81, p<0.001), and secondary education (OR = 2.59, 95% CI = 1.77−3.78, p<0.001) experienced a higher degree of physical abuse than those having higher education. People who attended primary education (OR = 1.88, 95% CI = 1.27−2.77, p = 0.002) and secondary education (OR = 2.04, 95% CI = 1.36−3.04, p<0.001) also experienced a higher degree of sexual abuse than those with higher education (see Table 5).

We revealed that respondents having no cash income (OR = 3.85, 95% CI = 1.56−9.51, p = 0.003) and earning less than US$30 (OR = 1.98, 95% CI = 1.24−3.16, p = 0.004) experienced more emotional abuse than those who earned US$30 or more per month. People earning less than US$30 (OR = 1.88, 95% CI = 1.19−2.98, p = 0.007) also experienced more physical abuse than those earning US$30 or more per month. However, we did not find any significant differences between people having no income and those earning US$30 or more in the exposure to physical MV (see Table 5). Respondents from a nuclear family experienced more physical abuse than those belonging to an extended family (OR = 1.62, 95% CI = 1.18−2.21, p = 0.003) (see Table 5).

## Discussion

This study examines gender differences in MV among the matrilineal ethnic-minority *Garo*, the patrilineal ethnic-minority *Santal*, and the patrilineal mainstream *Bengali* communities in rural Bangladesh. It shows that MV is largely a gendered phenomenon in the country. The rates of MV appear to be varied, according to the gender and social organization of the ethnic communities. In general, men are not subject to abuse within the marriage, but women are extensively exposed to all types of abuse from their spouses. Only a few men experienced emotional MV, and almost no men were exposed to physical and/or sexual abuse from their wives. Men are also not exposed to poly-victimization. In contrast, women are largely exposed to different/severe types of MV and poly-victimization, regardless of their ethnicities. MV appears to be a regular symptom of women’s life. However, different types of MV against women were significantly low in the matrilineal *Garo* community compared to the patrilineal *Bengali* and *Santal* Communities. In contrast, *Garo* men experienced more MV than the *Bengali* and *Santal* men. Regardless of their gender, *Garo* respondents experienced less physical MV than both the *Bengali* and *Santal* respondents. On the other hand, both the ethnic minority *Garo* and *Santal* respondents experienced less sexual abuse than the *Bengali* respondents. It appears that how the ethnic community is socially organized influences these differences in MV. It seems that the patterns and levels of MV among the patrilineal *Bengalis* are more similar to the patrilineal *Santals* than the matrilineal *Garos*.

The findings of our study are consistent with a body of previous studies that show a very high prevalence of MV among women in Bangladesh [4, 38, 39]. These studies estimated the rates of women experiencing MV or men perpetrating such abuses against women [19, 40], whereas our study includes both women and men as the victims of MV. To our knowledge, this is the first study in the country, where both women and men are regarded as victims as well as perpetrators of MV. Here, our study shows quite notably that men are not the usual subjects of MV in rural Bangladesh. It also shows that women are widely vulnerable to severe marital sexual abuse (marital rape), indicating how women’s sexuality is extremely/violently controlled by their husbands. This might be related to the societal level of gender inequality, where women are used to accepting forced sex by their husbands as a duty to their conjugal life [13].

Earlier studies mostly incorporated data from married women as victims of emotional, physical, or sexual MV [4, 38]. Our study not only estimated the rates of both men’s and women’s exposure to these MV but also revealed the extent of mild and severe abuse. These findings are important since no former studies have examined the severity of different types of MV in Bangladesh. This also helps us to understand the gendered dimensions of different types of MV. We speculate that when men are the victims of MV (mostly emotional abuse), this could be a facet of women’s resistance or the escalation of marital conflict [2], but when women are the victims of MV, this may be a part of the patriarchal controlling behaviors of their husband [2].

Our study further indicates that men in patrilineal communities were rarely victims of abuse, while some men in the matrilineal society experienced emotional violence. We assume that the experience/perpetration of MV in the various social organizations may be different, as the roles of men and women are different in these diverse ethnicities, as is the power dynamics.

**Fig 1:**
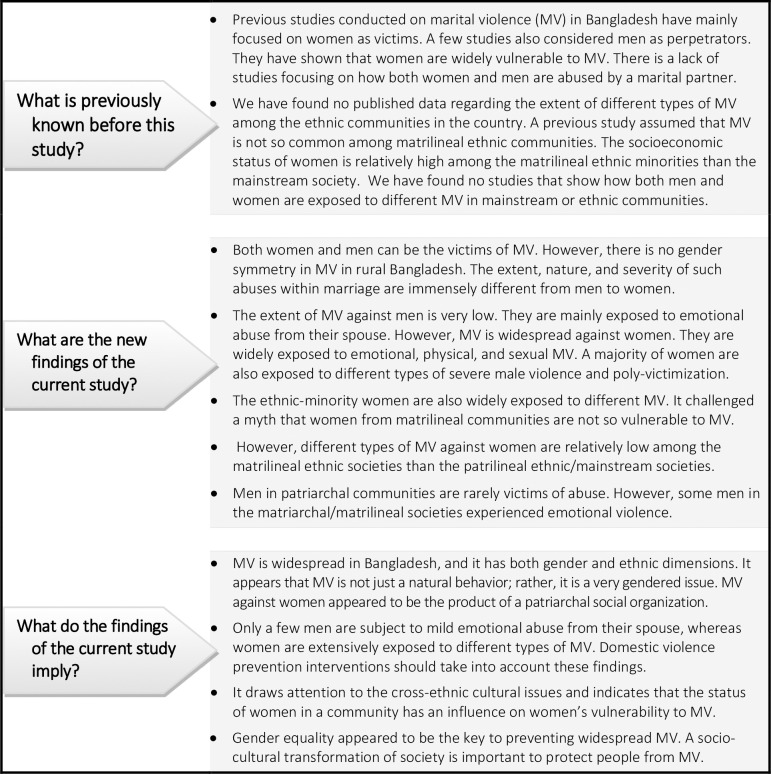
Significance of the current study in Bangladesh context.

Previous studies have rarely assessed the rates of MV among ethnic communities in Bangladesh. In that sense, our study contributes to the literature with further knowledge (see Fig 1). It shows that both the ethnic communities in our study sites differ from the mainstream *Bengali* community regarding their exposure to different types of MV. Although the magnitude and intensity of MV are higher among the *Bengali*, the findings reveal that women from ethnic minority communities also experience very high rates of MV from their husbands. These findings challenged a myth that the matrilineal society may not experience MV against women [23]. Nonetheless, our study indicates that the rates of severe MV are relatively low among the *Garo* community. These features of MV might be related to the gender construct of a matrilineal society, where women’s status is rather high compared to the mainstream community [13].

Broadly, the findings of our study support the idea that MV is a gender issue in rural Bangladesh, where women are primarily exposed to such abuses due to their subordinated position [20, 23, 41]. We believe that the gender regime of a community influences the nature of MV. Our study reveals that men are not subjects (victims) of MV in patriarchal societies, and the nature of MV against women can also be varied according to the gendered social structure of the community. The rates of MV among women are significantly lower among the ethnic minority *Garo* community than the *Bengali* community. This is probably because of their social structure/gender construct, where the *Garo* women are not only highly esteemed, but their socioeconomic status appeared to be rather high than in the *Bengali* community [13].

### Future research directions

We incorporated an adequate number of observations from the ethnic communities; however, the findings might be limited to that specific locality. Future studies should include other ethnic minority communities from diverse locations. The current findings are based on a single (individual) level analysis, although regional and village level variances may influence the individual-level estimates. A multilevel regression analysis appears appropriate for modeling the hierarchical data. Nevertheless, we lack the right design and suitable data to conduct such multilevel regression analysis in the current study. Although the current study is the first in the Bangladesh context, it may not be possible to properly understand the gender symmetry of such MV with these findings. As suggested by Michael P. Johnson [2], future studies should consider the different categorizations of MV, e.g., intimate terrorism, violent resistance, and situational couple violence. The gendered features of MV may vary according to the intention of abuse, such as whether the abuse is perpetrated to coercively control or dominate the partner (intimate terrorism); for defending oneself and/or fighting back against the abusive act (violence resistance); or whether the abuse occurs suddenly out of anger or frustration during an argument, without an intent to control (situational couple violence) [2].

### Conclusion

People’s exposure to abusive behaviors within marriage is very high in rural Bangladesh. However, our findings suggest that MV is not just a natural human behavior; rather, it is a gendered issue in the country. MV also has an ethnic dimension. The study shows that women are widely exposed to different types of MV in all ethnic communities. However, we also observed that the matrilineal ethnic minority *Garo* society is relatively less abusive toward women compared to both the patrilineal/patriarchal ethnic minority *Santal* and the mainstream *Bengali* ethnic communities. We further revealed that men are not generally subject to MV in patriarchal societies, although a few of them experienced emotional MV in the matrilineal society. It appears that the gendered structure of society influences the prevalence of MV. These findings indicate that gender equality may prevent marital abuses in society.

### Recommendations

Violence within the marital relationship is widespread in Bangladesh, and it has a gender and ethnocultural dimension. Here, we believe that addressing gendered socio-cultural issues is imperative to prevent the pervasive nature of MV in the patriarchal communities of the country. Although both women and men could be the victims of MV, the nature/extent of being exposed to MV significantly varies from women to men. Our study showed that only a few men are subject to mild-emotional victimization from their wives, whereas a majority of women are exposed to different types of severe MV from their husbands. Thus, domestic violence prevention interventions should take these findings into account. In order to raise public awareness as well as to initiate social action to prevent MV against women, the findings of the study may serve as a tool for advocacy and mass campaign. The messages of the study can be carved out for public consumption.

Besides the very high prevalence of emotional and physical MV, our study unveils that women are extremely vulnerable to severe sexual abuse, while society does not consider ‘male forced sex’ within the marriage as a rape crime. We also observe that societal-level factors such as gender construction (social organization of the community) have an immense influence on the extent/severity of MV in a community. Women are exposed to marital violence in all societies and ethnicities, while men are not exposed to such spousal abuse in the societies and ethnicities having patrilineal or patriarchal social organization. Women from matrilineal societies also experienced less marital violence than patrilineal or patriarchal societies. Thus, we believe that gender equality is the key to preventing widespread, as well as the gendered nature of marital victimization. The study indicates that marriage would be safer, particularly for women, if society can uphold a gender-sensitive socio-cultural mechanism. It also highlights that the socioeconomic status of women in a community has a great influence on women’s vulnerability to marital abuses. Hence, we argue that a comprehensive socio-cultural transformation of the patriarchal societies into a gender-equal order is very important for protecting people from widespread MV in the country.

Societal level initiatives should be undertaken in order to enhance women’s status and rights in society. Gender equality is also needed in matrilineal societies. This may include both women’s and men’s equal rights to inherit properties and their social mobility. The state should also criminalize marital rape, along with emotional and physical MV against women.

Our study also reveals that a higher level of education and personal income may prevent MV. Education may enlighten people, resulting in more self-understanding and respect toward others. Income may also enhance people’s dignity. People with higher income may also appraise less stress and frustration. Thus, increased income can prevent MV in different ways. We believe that socioeconomic development initiatives aimed to enhance gender equality through increasing income and education may primarily prevent different types of marital abuse in the country.

## Supporting information

S1 Fig Predicted plots of different types of MV by gender and ethnicity. (TIF)

S1 Table Sample profile by gender and ethnicity, results of χ^2^ tests and residual analysis. (PDF)

S2 Table MV by gender and ethnicity, 95% CI, results of χ^2^ tests and residual analysis. (PDF)

S1 File The survey questionnaire (*Bengali* and *English* versions). (PDF)

S2 File List of domestic violence support organizations/networks close to the study sites. (PDF)

S3 File Dataset. (SAV)
