# Supplementary figures and images for: Gender differences in marital violence: A cross-ethnic study among Bengali, Garo, and Santal communities in rural Bangladesh
Source: PLoS One. 2021 May 19;16(5):e0251574. doi: 10.1371/journal.pone.0251574 (PMC8133476; doi:10.1371/journal.pone.0251574)

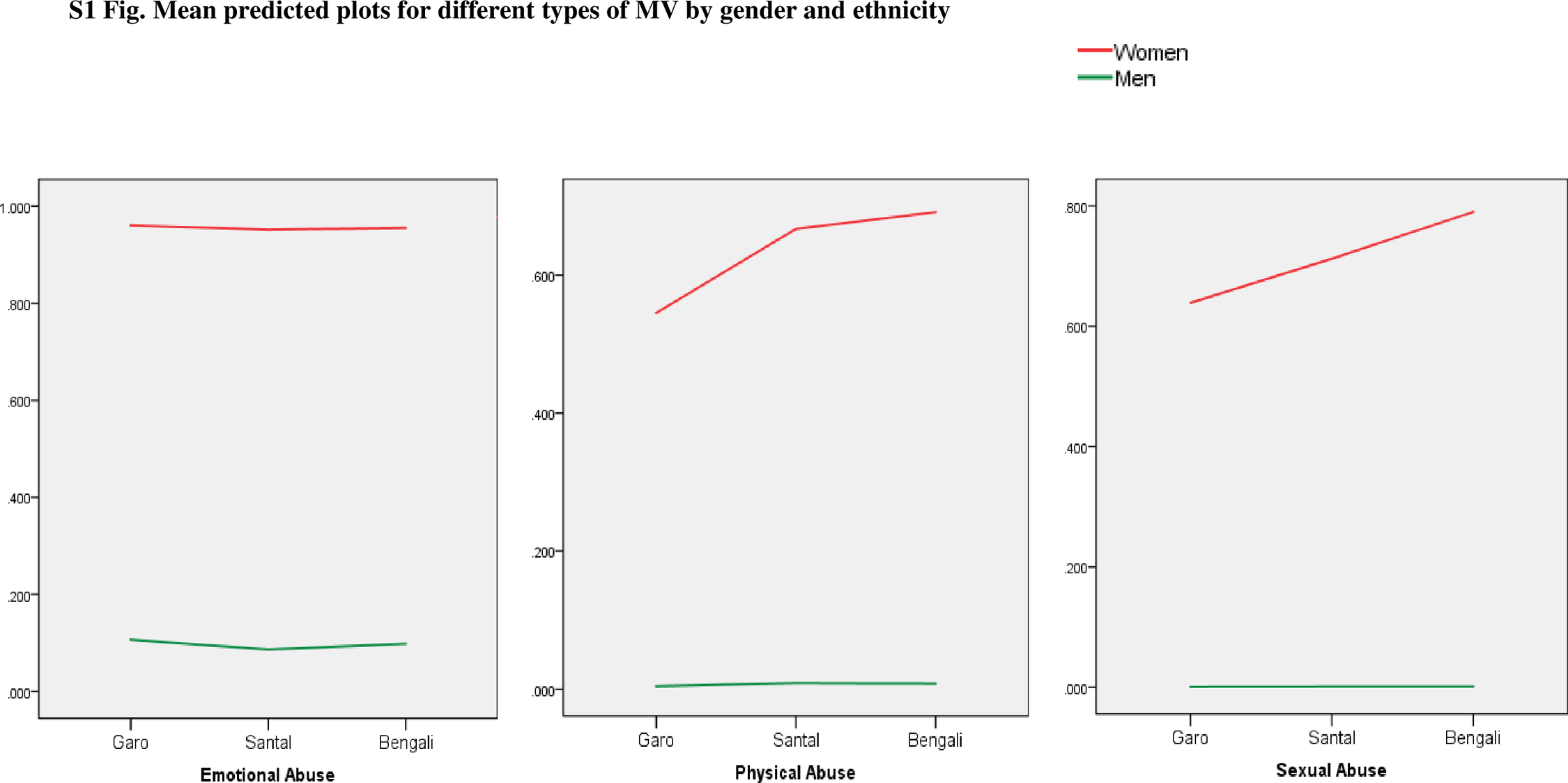

Supplement: S1 Fig — (TIF) [file pone.0251574.s001.tif]
