# Supplementary material for: Gender differences in marital violence: A cross-ethnic study among Bengali, Garo, and Santal communities in rural Bangladesh
Source: PLoS One. 2021 May 19;16(5):e0251574. doi: 10.1371/journal.pone.0251574 (PMC8133476; doi:10.1371/journal.pone.0251574)
Supplement: S1 Table — (PDF) [file pone.0251574.s002.pdf]

S1 Table. Sample profile by gender and ethnicity, results of χ^2 tests and residual analysis

Demographic profile and socio-economic status of the study participants by gender and ethnicity

	Overall				Men				Women			
	Total	Garo	Santal	Bengali	Total	Garo	Santal	Bengali	Total	Garo	Santal	Bengali
	N =1929 (%)	N=640 (%)	N= 640 (%)	N=649 (%)	n= 960 (%)	n=318 (%)	*n*=319 (%)	n=323 (%)	n=969 (%)	n=322 (%)	n=321 (%)	n=326 (%)
**Age in years**												
16-25	301 (15.6)	71 (11.1)	127 (19.8)	103 (15.9)	34 (3.5)	8 (2.5)	17 (5.3)	17 (5.3)	267 (27.6)	63 (19.6)	110 (34.3)	110 (34.3)
26–35	746 (38.7)	220 (34.4)	273 (42.7)	253 (39.0)	300 (31.3)	82 (25.8)	117 (36.7)	117 (36.7)	446 (46.0)	138 (42.8)	156 (48.6)	156 (48.6)
36–45	711 (36.8)	230 (35.9)	212 (33.1)	269 (41.4)	487 (50.7)	140 (44.0)	157 (49.2)	157 (49.2)	224 (23.1)	90 (28.0)	55 (17.1)	55 (17.1)
46–60	171 (8.9)	119 (18.6)	28 (4.4)	24 (3.7)	139 (14.5)	88 (27.7)	28 (8.8)	28 (8.8)	32 (3.3)	31 (9.6)	0 (0.0)	0 (0.0)
**Years married**												
1-10	679 (35.2)	225 (35.2)	260 (40.6)	194 (29.9)	267 (27.8)	87 (27.4)	98 (30.7)	82 (25.4)	412 (42.5)	138 (42.8)	162 (50.5)	112 (34.4)
11-20	904 (46.9)	244 (38.1)	319 (49.9)	341 (52.5)	531 (55.3)	144 (45.3)	187 (58.6)	200 (61.9)	373 (38.5)	100 (31.1)	132 (41.1)	141 (43.2)
21-38	346 (17.9)	171 (26.7)	61 (9.5)	114 (17.6)	162 (16.9)	87 (27.3)	34 (10.7)	41 (12.7)	184 (19.0)	84 (26.1)	27 (8.4)	73 (22.4)
**Education**												
No schooling	149 (7.7)	28 (4.4)	76 (11.9)	45 (6.9)	51 (5.3)	13 (4.1)	27 (8.5)	11 (3.4)	98 (10.1)	15 (4.7)	49 (15.3)	34 (10.4)
Primary	820 (42.5)	278 (43.4)	304 (47.5)	238 (36.7)	457 (47.6)	161 (50.6)	171 (53.6)	125 (38.7)	363 (37.5)	117 (36.3)	133 (41.4)	113 (34.7)
Secondary	575 (29.8)	188 (29.4)	166 (25.9)	221 (34.1)	228 (30.0)	94 (29.6)	90 (28.2)	104 (32.2)	287 (29.6)	94 (29.2)	76 (23.7)	117 (35.9)
Higher	385 (20.0)	146 (22.8)	94 (14.7)	145 (22.3)	164 (17.1)	50 (15.7)	31 (9.7)	83 (25.7)	221 (22.8)	96 (29.8)	63 (19.6)	62 (19.0)
**Main occupation**												
Home-making	636 (33.0)	167 (26.1)	153 (23.9)	316 (48.7)	3 (0.3)	3 (0.9)	0 (0.0)	0 (0.0)	633 (65.3)	164 (50.9)	153 (47.7)	316 (97.0)
Day laborers	797 (41.3)	265 (41.4)	435 (68.0)	97 (14.9)	537 (55.9)	164 (51.6)	278 (87.2)	95 (29.4)	260 (26.8)	101 (31.4)	157 (48.9)	2 (0.6)
Agric farming	329 (17.0)	154 (24.1)	29 (4.5)	146 (22.5)	281 (29.3)	116 (36.5)	25 (7.8)	140 (43.3)	48 (5.0)	38 (11.8)	4 (1.2)	6 (1.8)
Job and others	167 (8.7)	54 (8.4)	23 (3.6)	90 (13.9)	139 (14.5)	35 (11.0)	16 (5.0)	88 (27.3)	28 (2.9)	19 (5.9)	7 (2.2)	2 (0.6)
**Monthly income**												
No income	423 (21.9)	101 (15.8)	119 (18.6)	203 (31.3)	2 (0.2)	2 (0.6)	0 (0.0)	0 (0.0)	421 (43.4)	99 (30.7)	119 (37.1)	203 (62.2)
Less than $30	575 (29.8)	134 (20.9)	289 (45.2)	152 (23.4)	177 (18.4)	15 (4.7)	122 (38.2)	40 (12.4)	398 (41.1)	119 (37.0)	167 (52.0)	112 (34.4)
$30 and above	931 (48.3)	405 (63.3)	232 (36.2)	294 (45.3)	781 (81.4)	301 (94.7)	197 (61.8)	283 (87.6)	150 (15.5)	104 (32.3)	35 (10.9)	11 (3.4)
**Family structure**												
Nuclear	1411 (73.1)	394 (61.6)	511 (79.8)	506 (78.0)	738 (76.9)	206 (64.8)	274 (85.9)	258 (79.9)	673 (69.5)	188 (58.4)	237 (73.8)	248 (76.1)
Extended	518 (26.9)	246 (38.4)	129 (20.2)	143 (22.0)	222 (23.1)	112 (35.2)	45 (14.1)	65 (20.1)	296 (30.5)	134 (41.6)	84 (26.2)	78 (23.9)

Results of χ^2 tests and post hoc (residual) analysis

Socioeconomic status by ethnicity

			Ethnicity			
			Garo	Santal	Bengali	Total
**Education**	Upper	Count	146	94	145	385
		% within Ethnicity	22.8%	14.7%	22.3%	20.0%
		Standardized Residual	1.6	-3.0	1.4	
	Secondary	Count	188	166	221	575
		% within Ethnicity	29.4%	25.9%	34.1%	29.8%
		Standardized Residual	-.2	-1.8	2.0	
	Primary	Count	278	304	238	820
		% within Ethnicity	43.4%	47.5%	36.7%	42.5%
		Standardized Residual	.4	1.9	-2.3	
	None	Count	28	76	45	149
		% within Ethnicity	4.4%	11.9%	6.9%	7.7%
		Standardized Residual	-3.0	3.8	-.7	
Total		Count	640	640	649	1929
		% within Ethnicity	100.0%	100.0%	100.0%	100.0%

χ^2=53.724, df=6, p<.001

			Ethnicity			
			Garo	Santal	Bengali	Total
**Main occupation**	Home-making	Count	167	153	316	636
		% within Ethnicity	26.1%	23.9%	48.7%	33.0%
		Standardized Residual	-3.0	-4.0	7.0	
	Agric farming	Count	154	29	146	329
		% within Ethnicity	24.1%	4.5%	22.5%	17.1%
		Standardized Residual	4.3	-7.7	3.4	
	Day laborers	Count	265	435	97	797
		% within Ethnicity	41.4%	68.0%	14.9%	41.3%
		Standardized Residual	.0	10.5	-10.5	
	Job and others	Count	54	23	90	167
		% within Ethnicity	8.4%	3.6%	13.9%	8.7%
		Standardized Residual	-.2	-4.4	4.5	
Total		Count	640	640	649	1929
		% within Ethnicity	100.0%	100.0%	100.0%	100.0%

χ^2=420.925, df=6, p<.001

			Ethnicity			
			Garo	Santal	Bengali	Total
**Monthly income**	No income	Count	101	119	203	423
		% within Ethnicity	15.8%	18.6%	31.3%	21.9%
		Standardized Residual	-3.3	-1.8	5.1	
	Less than $30	Count	134	289	152	575
		% within Ethnicity	20.9%	45.2%	23.4%	29.8%
		Standardized Residual	-4.1	7.1	-3.0	
	$30 and above	Count	405	232	294	931
		% within Ethnicity	63.3%	36.3%	45.3%	48.3%
		Standardized Residual	5.5	-4.4	-1.1	
Total		Count	640	640	649	1929
		% within Ethnicity	100.0%	100.0%	100.0%	100.0%

χ^2=166.731, df=4, p<.001

			Ethnicity			
			Garo	Santal	Bengali	Total
**Family structure**	Nuclear	Count	394	511	506	1411
		% within Ethnicity	61.6%	79.8%	78.0%	73.1%
		Standardized Residual	-3.4	2.0	1.4	
	Joint	Count	246	129	143	518
		% within Ethnicity	38.4%	20.2%	22.0%	26.9%
		Standardized Residual	5.7	-3.3	-2.4	
Total		Count	640	640	649	1929
		% within Ethnicity	100.0%	100.0%	100.0%	100.0%

χ^2=66.012, df=2, p<.001

Socioeconomic status among women by ethnicity

			Ethnicity			Total
			Garo	Santal	Bengali	
**Schooling**	Upper	Count	96	63	62	221
		% within Ethnicity	29.8%	19.6%	19.0%	22.8%
		Standardized Residual	2.6	-1.2	-1.4	
	Secondary	Count	94	76	117	287
		% within Ethnicity	29.2%	23.7%	35.9%	29.6%
		Standardized Residual	-.1	-2.0	2.1	
	Primary	Count	117	133	113	363
		% within Ethnicity	36.3%	41.4%	34.7%	37.5%
		Standardized Residual	-.3	1.2	-.8	
	None	Count	15	49	34	98
		% within Ethnicity	4.7%	15.3%	10.4%	10.1%
		Standardized Residual	-3.1	2.9	.2	
Total		Count	322	321	326	969
		% within Ethnicity	100.0%	100.0%	100.0%	100.0%
a. Sex = Women						

χ^2=38.654, df=6, p<.001

			Ethnicity			Total
			Garo	Santal	Bengali	
**Main occupation**	Home-making	Count	164	153	316	633
		% within Ethnicity	50.9%	47.7%	96.9%	65.3%
		Standardized Residual	-3.2	-3.9	7.1	
	Agric farming	Count	38	4	6	48
		% within Ethnicity	11.8%	1.2%	1.8%	5.0%
		Standardized Residual	5.5	-3.0	-2.5	
	Day laborers	Count	101	157	2	260
		% within Ethnicity	31.4%	48.9%	0.6%	26.8%
		Standardized Residual	1.6	7.6	-9.1	
	Job and others	Count	19	7	2	28
		% within Ethnicity	5.9%	2.2%	0.6%	2.9%
		Standardized Residual	3.2	-.7	-2.4	
Total		Count	322	321	326	969
		% within Ethnicity	100.0%	100.0%	100.0%	100.0%
a. Sex = Women						

χ^2=281.966, df=6, p<.001

			Ethnicity			Total
			Garo	Santal	Bengali	
**Monthly income**	No income	Count	99	119	203	421
		% within Ethnicity	30.7%	37.1%	62.3%	43.4%
		Standardized Residual	-3.5	-1.7	5.2	
	Less than $30	Count	119	167	112	398
		% within Ethnicity	37.0%	52.0%	34.4%	41.1%
		Standardized Residual	-1.2	3.1	-1.9	
	$30 and above	Count	104	35	11	150
		% within Ethnicity	32.3%	10.9%	3.4%	15.5%
		Standardized Residual	7.7	-2.1	-5.6	
Total		Count	322	321	326	969
		% within Ethnicity	100.0%	100.0%	100.0%	100.0%
a. Sex = Women						

χ^2=149.871, df=4, p<.001

Crosstab[a]

			Ethnicity			Total
			Garo	Santal	Bengali	
**Family structure**	Nuclear	Count	188	237	248	673
		% within Ethnicity	58.4%	73.8%	76.1%	69.5%
		Standardized Residual	-2.4	.9	1.4	
	Joint	Count	134	84	78	296
		% within Ethnicity	41.6%	26.2%	23.9%	30.5%
		Standardized Residual	3.6	-1.4	-2.2	
Total		Count	322	321	326	969
		% within Ethnicity	100.0%	100.0%	100.0%	100.0%
a. Sex = Women						

χ^2=28.228, df=2, p<.001

Socioeconomic status among men by ethnicity

			Ethnicity			Total
			Garo	Santal	Bengali	
**Education**	Upper	Count	50	31	83	164
		% within Ethnicity	15.7%	9.7%	25.7%	17.1%
		Standardized Residual	-.6	-3.2	3.7	
	Secondary	Count	94	90	104	288
		% within Ethnicity	29.6%	28.2%	32.2%	30.0%
		Standardized Residual	-.1	-.6	.7	
	Primary	Count	161	171	125	457
		% within Ethnicity	50.6%	53.6%	38.7%	47.6%
		Standardized Residual	.8	1.6	-2.3	
	None	Count	13	27	11	51
		% within Ethnicity	4.1%	8.5%	3.4%	5.3%
		Standardized Residual	-.9	2.4	-1.5	
Total		Count	318	319	323	960
		% within Ethnicity	100.0%	100.0%	100.0%	100.0%
a. Sex = Men						

χ^2=42.004, df=6, p<.001

			Ethnicity			Total
			Garo	Santal	Bengali	
**Main occupation**	Home-making	Count	3	0	0	3
		% within Ethnicity	0.9%	0.0%	0.0%	0.3%
		Standardized Residual	2.0	-1.0	-1.0	
	Agric farming	Count	116	25	140	281
		% within Ethnicity	36.5%	7.8%	43.3%	29.3%
		Standardized Residual	2.4	-7.1	4.7	
	Day laborers	Count	164	278	95	537
		% within Ethnicity	51.6%	87.1%	29.4%	55.9%
		Standardized Residual	-1.0	7.5	-6.4	
	Job and others	Count	35	16	88	139
		% within Ethnicity	11.0%	5.0%	27.2%	14.5%
		Standardized Residual	-1.6	-4.4	6.0	
Total		Count	318	319	323	960
		% within Ethnicity	100.0%	100.0%	100.0%	100.0%
a. Sex = Men						

χ^2=239.613, df=6, p<.001

			Ethnicity			Total
			Garo	Santal	Bengali	
**Monthly income**	No income	Count	2	0	0	2
		% within Ethnicity	0.6%	0.0%	0.0%	0.2%
		Standardized Residual	1.6	-.8	-.8	
	Less than $30	Count	15	122	40	177
		% within Ethnicity	4.7%	38.2%	12.4%	18.4%
		Standardized Residual	-5.7	8.2	-2.5	
	$30 and above	Count	301	197	283	781
		% within Ethnicity	94.7%	61.8%	87.6%	81.4%
		Standardized Residual	2.6	-3.9	1.2	
Total		Count	318	319	323	960
		% within Ethnicity	100.0%	100.0%	100.0%	100.0%
a. Sex = Men						

χ^2=134.337, df=4, p<.001

			Ethnicity			Total
			Garo	Santal	Bengali	
**Family structure**	Nuclear	Count	206	274	258	738
		% within Ethnicity	64.8%	85.9%	79.9%	76.9%
		Standardized Residual	-2.5	1.8	.6	
	Joint	Count	112	45	65	222
		% within Ethnicity	35.2%	14.1%	20.1%	23.1%
		Standardized Residual	4.5	-3.3	-1.1	
Total		Count	318	319	323	960
		% within Ethnicity	100.0%	100.0%	100.0%	100.0%
a. Sex = Men						

χ^2=42.399, df=2, p<.001
