# Supplementary material for: Gender differences in marital violence: A cross-ethnic study among Bengali, Garo, and Santal communities in rural Bangladesh
Source: PLoS One. 2021 May 19;16(5):e0251574. doi: 10.1371/journal.pone.0251574 (PMC8133476; doi:10.1371/journal.pone.0251574)
Supplement: S2 Table — (PDF) [file pone.0251574.s003.pdf]

S2 Table. MV by gender and ethnicity, 95% CI, results of χ^2 tests and residual analysis

The prevalence of lifetime experience of different types of marital violence by gender and ethnicity

	Overall				Men				Women			
	Total	Garo	Santal	Bengali	Total	Garo	Santal	Bengali	Total	Garo	Santal	Bengali
	N =1929 (%) (95% CI)	N=640 (%) (95% CI)	N= 640 (%) (95% CI)	N=649 (%) (95% CI)	n= 960 (%) (95% CI)	n=318 (%) (95% CI)	*n*=319 (%) (95% CI)	n=323 (%) (95% CI)	n=969 (%) (95% CI)	n=322 (%) (95% CI)	n=321 (%) (95% CI)	n=326 (%) (95% CI)
**Emotional violence**												
No	910 (47.2) (45.0–49.4)	297 (46.4) (42.8–50.3)	307 (48.0) (44.1–51.7)	306 (47.1) (43.3–51.2)	867 (90.3) (88.3–92.2)	267 (84.0) (79.6–87.4)	305 (95.6) (93.1–97.8)	295 (91.3) (88.2–94.4)	43 (4.4) (3.2–5.8)	30 (9.3) (6.2–12.7)	2 (0.6) (0.0–1.6)	11 (3.4) (1.5–5.5)
Yes	1019 (52.8) (50.6–55.0)	343 (53.6) (49.7–57.2)	333 (52.0) (48.3–55.9)	343 (52.9) (48.8–56.7)	93 (9.7) (7.8–11.7)	51 (16.0) (12.6–20.4)	14 (4.4) (2.2–6.9)	28 (8.7) (5.6–11.8)	926 (95.6) (94.2–96.8)	292 (90.7) (87.3–93.8)	319 (99.4) (98.4–100.0)	315 (96.6) (94.5–98.5)
Mild	431 (22.3) (20.5–24.1)	182 (28.4) (25.0–31.9)	136 (21.3) (18.1–24.4)	113 (17.4) (14.5–20.5)	61 (6.4) (4.8–7.9)	32 (10.1) (7.2–13.5)	12 (3.8) (1.9–6.0)	17 (5.3) (2.8–7.7)	370 (38.2) (35.1–41.3)	150 (46.6) (41.0–52.2)	124 (38.6) (33.3–44.2)	96 (29.4) (25.2–34.4)
Severe	588 (30.5) (28.4–32.5)	161 (25.2) (22.0–28.6)	197 (30.8) (27.3–34.5)	230 (35.4) (31.7–39.1)	32 (3.3) (2.2–4.6)	19 (6.0) (3.5–8.8)	2 (0.6) (0.0–1.6)	11 (03.4) (1.5–5.6)	556 (57.4) (54.3–60.8)	142 (44.1) (38.5–49.4)	195 (60.7) (55.1–66.0)	219 (67.2) (62.0–71.8)
**Physical violence**												
No	1307 (67.8) (65.6–70.0)	463 (72.3) (68.9–75.6)	423 (66.1) (62.2–69.8)	421 (64.9) (61.3–68.6)	953 (99.3) (98.6–99.8)	313 (98.4) (96.9–99.7)	318 (99.7) (98.7–100.0)	322 (99.7) (99.1–100.0)	354 (36.5) (33.5–39.6)	150 (46.6) (41.3–52.5)	105 (32.7) (27.7–37.7)	99 (30.4) (25.5–35.6)
Yes	622 (32.2) (30.0–34.4)	177 (27.7) (24.4–31.1)	217 (33.9) (30.2–37.8)	228 (35.1) (31.4–38.7)	7 (0.7) (0.2–1.4)	5 (1.6) (0.3–3.1)	1 (0.3) (0.0–1.3)	1 (0.3) (0.0–0.9)	615 (63.5) (60.4–66.5)	172 (53.4) (47.5–58.7)	216 (67.3) (62.3–72.3)	227 (69.6) (64.4–74.5)
Mild	281 (14.6) (13.0–16.1)	70 (10.9) (8.4–13.6)	99 (15.5) (12.8–18.3)	112 (17.3) (14.3–20.3)	5 (0.5) (0.1–1.0)	3 (0.9) (0.0–2.2)	1 (0.3) (0.0–1.3)	1 (0.3) (0.0–0.9)	276 (28.5) (25.7–31.3)	67 (20.8) (16.5–25.2)	98 (30.5) (25.9–35.5)	111 (34.0) (29.1–39.0)
Severe	341 (17.7) (16.0–19.2)	107 (16.7) (13.8–19.8)	118 (18.4) (15.5–21.4)	116 (17.9) (14.8–20.8)	2 (0.2) (0.0–0.5)	2 (0.6) (0.0–1.6)	0 (0.0) (0.0–0.0)	0 (0.0) (0.0–0.0)	339 (35.0) (32.1–38.1)	105 (32.6) (27.6–38.2)	118 (36.8) (31.8–41.7)	116 (35.6) (30.4–40.8)
**Sexual violence**												
No	1236 (64.1) (62.1–66.2)	434 (67.8) (64.2–71.4)	411 (64.2) (60.6–67.8)	391 (60.2) (56.4–63.9)	959 (99.9) (99.6–100.0)	318 (100.0) (100.0–100.0)	319 (100.0) (100.0–100.0)	322 (99.7) (99.1–100.0)	277 (28.6) (25.6–31.5)	116 (36.0) (31.1–41.6)	92 (28.7) (23.7–33.6)	69 (21.2) (17.2–25.5)
Yes	693 (35.9) (33.8–37.9)	206 (32.2) (28.6–35.8)	229 (35.8) (32.2–39.4)	258 (39.8) (36.1–43.6)	1 (0.1) (0.0–0.4)	0 (0.0) (0.0–0.0)	0 (0.0) (0.0–0.0)	1 (0.3) (0.0–0.9)	692 (71.4) (68.5–74.4)	206 (64.0) (58.4–68.9)	229 (71.3) (66.4–76.3)	257 (78.8) (74.5–82.8)
Mild	63 (3.3) (2.4–4.0)	22 (3.4) (2.0–5.0)	20 (3.1) (1.9–4.5)	21 (3.2) (1.8–4.6)	1 (00.1) (0.0–0.4)	0 (0.0) (0.0–0.0)	0 (0.0) (0.0–0.0)	1 (0.3) (0.0–0.9)	62 (6.4) (4.9–8.0)	22 (6.8) (4.0–9.6)	20 (6.2) (3.7–9.0)	20 (6.1) (3.7–8.9)
Severe	630 (32.7) (30.6–34.6)	184 (28.7) (25.3–32.3)	209 (32.7) (29.2–36.1)	237 (36.5) (33.0–40.4)	0 (0.0) (0.0–0.0)	0 (0.0) (0.0–0.0)	0 (0.0) (0.0–0.0)	0 (0.0) (0.0–0.0)	630 (65.0) (61.9–68.0)	184 (57.1) (51.6–62.4)	209 (65.1) (59.8–70.4)	237 (72.7) (67.5–77.3)
**Overall violence**												
**Any type (yes)**	1033 (53.6) (51.3–55.7)	351 (54.8) (50.9–58.4)	333 (52.0) (48.3–55.9)	349 (53.8) (49.9–57.6)	95 (9.9) (8.0–11.9)	52 (16.4) (12.9–20.8)	14 (4.4) (2.2–6.9)	29 (9.0) (5.9–12.1)	938 (96.8) (95.8–97.8)	299 (92.9) (89.8–95.3)	319 (99.4) (98.4–100.0)	320 (98.2) (96.3–99.4)
**Poly abuse (yes)**	490 (25.4) (23.4–27.2)	125 (19.5) (16.6–22.7)	173 (27.0) (23.6–30.5)	192 (29.6) (26.2–33.0)	0 (0.0) (0.0–0.0)	0 (0.0) (0.0–0.0)	0 (0.0) (0.0–0.0)	0 (0.0) (0.0–0.0)	490 (50.6) (47.4–53.4)	125 (38.8) (33.5–44.1)	173 (53.9) (48.9–59.2)	192 (58.9) (53.4–64.1)

Note: 95% CI bootstrap results are based on 1000 bootstrap samples

Results of χ^2 tests and post hoc (residual) analysis

Experience of MV by ethnicity

			Ethnicity			
			Garo	Santal	Bengali	Total
**Ever marital emotional**	None	Count	297	307	306	910
		% within Ethnicity	46.4%	48.0%	47.1%	47.2%
		Standardized Residual	-.3	.3	.0	
	Mild	Count	182	136	113	431
		% within Ethnicity	28.4%	21.3%	17.4%	22.3%
		Standardized Residual	3.3	-.6	-2.7	
	Severe	Count	161	197	230	588
		% within Ethnicity	25.2%	30.8%	35.4%	30.5%
		Standardized Residual	-2.4	.1	2.3	
Total		Count	640	640	649	1929
		% within Ethnicity	100.0%	100.0%	100.0%	100.0%

χ^2=29.41, df=4, p<.001

			Ethnicity			
			Garo	Santal	Bengali	Total
**Ever marital physical**	None	Count	463	423	421	1307
		% within Ethnicity	72.3%	66.1%	64.9%	67.8%
		Standardized Residual	1.4	-.5	-.9	
	Mild	Count	70	99	112	281
		% within Ethnicity	10.9%	15.5%	17.3%	14.6%
		Standardized Residual	-2.4	.6	1.8	
	Severe	Count	107	118	116	341
		% within Ethnicity	16.7%	18.4%	17.9%	17.7%
		Standardized Residual	-.6	.5	.1	
Total		Count	640	640	649	1929
		% within Ethnicity	100.0%	100.0%	100.0%	100.0%

χ^2=12.973, df=4, p=.011

			Ethnicity			Total
			Garo	Santal	Bengali	
**Ever severe sexual severity**	None	Count	434	411	391	1236
		% within Ethnicity	67.8%	64.2%	60.2%	64.1%
		Standardized Residual	1.2	.0	-1.2	
	Mild	Count	22	20	21	63
		% within Ethnicity	3.4%	3.1%	3.2%	3.3%
		Standardized Residual	.2	-.2	.0	
	Severe	Count	184	209	237	630
		% within Ethnicity	28.7%	32.7%	36.5%	32.7%
		Standardized Residual	-1.7	.0	1.7	
Total		Count	640	640	649	1929
		% within Ethnicity	100.0%	100.0%	100.0%	100.0%

χ^2=8.934, df=4, p=.063

			Ethnicity			Total
			Garo	Santal	Bengali	
**Ever marital any abuse**	No	Count	289	307	300	896
		% within Ethnicity	45.2%	48.0%	46.2%	46.4%
		Standardized Residual	-.5	.6	-.1	
	Yes	Count	351	333	349	1033
		% within Ethnicity	54.8%	52.0%	53.8%	53.6%
		Standardized Residual	.4	-.5	.1	
Total		Count	640	640	649	1929
		% within Ethnicity	100.0%	100.0%	100.0%	100.0%

χ^2=1.037, df=2, p=.595

			Ethnicity			Total
			Garo	Santal	Bengali	
**Ever marital poly abuse**	No	Count	515	467	457	1439
		% within Ethnicity	80.5%	73.0%	70.4%	74.6%
		Standardized Residual	1.7	-.5	-1.2	
	Yes	Count	125	173	192	490
		% within Ethnicity	19.5%	27.0%	29.6%	25.4%
		Standardized Residual	-2.9	.8	2.1	
Total		Count	640	640	649	1929
		% within Ethnicity	100.0%	100.0%	100.0%	100.0%

χ^2=18.527, df=2, p<.001

Experience of MV among women by ethnicity

			Ethnicity			Total
			Garo	Santal	Bengali	
Ever marital emotional severity	None	Count	30	2	11	43
		% within Ethnicity	9.3%	0.6%	3.4%	4.4%
		Standardized Residual	4.2	-3.2	-.9	
	Mild	Count	150	124	96	370
		% within Ethnicity	46.6%	38.6%	29.4%	38.2%
		Standardized Residual	2.4	.1	-2.6	
	Severe	Count	142	195	219	556
		% within Ethnicity	44.1%	60.7%	67.2%	57.4%
		Standardized Residual	-3.1	.8	2.3	
Total		Count	322	321	326	969
		% within Ethnicity	100.0%	100.0%	100.0%	100.0%
a. Sex = Women						

χ^2=57.100, df=4, p<.001

			Ethnicity			Total
			Garo	Santal	Bengali	
Ever marital physical severity	None	Count	150	105	99	354
		% within Ethnicity	46.6%	32.7%	30.4%	36.5%
		Standardized Residual	3.0	-1.1	-1.8	
	Mild	Count	67	98	111	276
		% within Ethnicity	20.8%	30.5%	34.0%	28.5%
		Standardized Residual	-2.6	.7	1.9	
	Severe	Count	105	118	116	339
		% within Ethnicity	32.6%	36.8%	35.6%	35.0%
		Standardized Residual	-.7	.5	.2	
Total		Count	322	321	326	969
		% within Ethnicity	100.0%	100.0%	100.0%	100.0%
a. Sex = Women						

χ^2=25.100, df=4, p<.001

			Ethnicity			Total
			Garo	Santal	Bengali	
**Ever severe sexual severity**	None	Count	116	92	69	277
		% within Ethnicity	36.0%	28.7%	21.2%	28.6%
		Standardized Residual	2.5	.0	-2.5	
	Mild	Count	22	20	20	62
		% within Ethnicity	6.8%	6.2%	6.1%	6.4%
		Standardized Residual	.3	-.1	-.2	
	Severe	Count	184	209	237	630
		% within Ethnicity	57.1%	65.1%	72.7%	65.0%
		Standardized Residual	-1.8	.0	1.7	
Total		Count	322	321	326	969
		% within Ethnicity	100.0%	100.0%	100.0%	100.0%

χ^2=18.688, df=4, p=.001

			Ethnicity			Total
			Garo	Santal	Bengali	
Ever marital any abuse	No	Count	23	2	6	31
		% within Ethnicity	7.1%	0.6%	1.8%	3.2%
		Standardized Residual	4.0	-2.6	-1.4	
	Yes	Count	299	319	320	938
		% within Ethnicity	92.9%	99.4%	98.2%	96.8%
		Standardized Residual	-.7	.5	.2	
Total		Count	322	321	326	969
		% within Ethnicity	100.0%	100.0%	100.0%	100.0%

χ^2=24.993, df=2, <=.001

			Ethnicity			Total
			Garo	Santal	Bengali	
Ever marital poly abuse	No	Count	197	148	134	479
		% within Ethnicity	61.2%	46.1%	41.1%	49.4%
		Standardized Residual	3.0	-.8	-2.1	
	Yes	Count	125	173	192	490
		% within Ethnicity	38.8%	53.9%	58.9%	50.6%
		Standardized Residual	-3.0	.8	2.1	
Total		Count	322	321	326	969
		% within Ethnicity	100.0%	100.0%	100.0%	100.0%

χ^2=28.244, df=2, <=.001

Experience of MV among men by ethnicity

			Ethnicity			
			Garo	Santal	Bengali	Total
Ever marital emotional severity	None	Count	267	305	295	867
		% within Ethnicity	84.0%	95.6%	91.3%	90.3%
		Standardized Residual	-1.2	1.0	.2	
	Mild	Count	32	12	17	61
		% within Ethnicity	10.1%	3.8%	5.3%	6.4%
		Standardized Residual	2.6	-1.8	-.8	
	Severe	Count	19	2	11	32
		% within Ethnicity	6.0%	0.6%	3.4%	3.3%
		Standardized Residual	2.6	-2.6	.1	
Total		Count	318	319	323	960
		% within Ethnicity	100.0%	100.0%	100.0%	100.0%
a. Sex = Men						

χ^2=26.983, df=4, p<.001

Crosstab[a]

			Ethnicity			
			Garo	Santal	Bengali	Total
**Ever marital physical severity**	None	Count	313	318	322	953
		% within Ethnicity	98.4%	99.7%	99.7%	99.3%
		Standardized Residual	-.2	.1	.1	
	Mild	Count	3	1	1	5
		% within Ethnicity	0.9%	0.3%	0.3%	0.5%
		Standardized Residual	1.0	-.5	-.5	
	Severe	Count	2	0	0	2
		% within Ethnicity	0.6%	0.0%	0.0%	0.2%
		Standardized Residual	1.6	-.8	-.8	
Total		Count	318	319	323	960
		% within Ethnicity	100.0%	100.0%	100.0%	100.0%
a. Sex = Men						

χ^2=5.702, df=4, p=.223

			Ethnicity			Total
			Garo	Santal	Bengali	
**Ever severe sexual severity**	None	Count	318	319	322	959
		% within Ethnicity	100.0%	100.0%	99.7%	99.9%
		Standardized Residual	.0	.0	.0	
	Mild	Count	0	0	1	1
		% within Ethnicity	0.0%	0.0%	0.3%	0.1%
		Standardized Residual	-.6	-.6	1.1	
Total		Count	318	319	323	960
		% within Ethnicity	100.0%	100.0%	100.0%	100.0%
a. Sex = Men						

χ^2=1.974, df=2, p=.373

			Ethnicity			Total
			Garo	Santal	Bengali	
**Ever marital any abuse**	No	Count	266	305	294	865
		% within Ethnicity	83.6%	95.6%	91.0%	90.1%
		Standardized Residual	-1.2	1.0	.2	
	Yes	Count	52	14	29	95
		% within Ethnicity	16.4%	4.4%	9.0%	9.9%
		Standardized Residual	3.7	-3.1	-.5	
Total		Count	318	319	323	960
		% within Ethnicity	100.0%	100.0%	100.0%	100.0%
a. Sex = Men						

χ^2=26.022, df=2, p<.001

			Ethnicity			Total
			Garo	Santal	Bengali	
**Ever marital poly abuse**	No	Count	318	319	323	960
		% within Ethnicity	100.0%	100.0%	100.0%	100.0%
		Standardized Residual	.0	.0	.0	
Total		Count	318	319	323	960
		% within Ethnicity	100.0%	100.0%	100.0%	100.0%
a. Sex = Men						
