# Supplementary material for: Gender differences in marital violence: A cross-ethnic study among Bengali, Garo, and Santal communities in rural Bangladesh
Source: PLoS One. 2021 May 19;16(5):e0251574. doi: 10.1371/journal.pone.0251574 (PMC8133476; doi:10.1371/journal.pone.0251574)
Supplement: S1 File — (PDF) [file pone.0251574.s004.pdf]

S1 File. The survey questionnaire (*Bengali* and *English* Versions)

Bengali Version

Socio-demographic Profile

১. এথনিসিটিঃ ১) বাঙালী, ২) সাঁওতাল, ৩) গারো
২. উত্তরদাতার লিঙ্গঃ ১) নারী, ২) পুরুষ
৩. বর্তমান বয়স কত বছর? ___________
৪. সঙ্গীর (স্বামী বা স্ত্রীর) বয়স কত বছর? ___________
৫. কত বছর বিয়ে হয়েছে? ___________
৬. আপনার সন্তান কত জন? ___________
৭. পরিবারের কাঠামোঃ ১) একক, ২) বর্ধিত
৮. শিক্ষাগত যোগ্যতা (কত বছর পর্যন্ত স্কুলে গিয়েছেন?) ___________
৯. সঙ্গীর (স্বামী বা স্ত্রীর) শিক্ষাগত যোগ্যতা (কত বছর পর্যন্ত স্কুলে গিয়েছেন?)ঃ ___________
১০. আপনার প্রধান পেশাঃ ১) গৃহকাজ/চাকুরী প্রার্থী, ২) কৃষিকাজ, ৩) রিক্সা/দিনমজুর, ৪) ব্যবসা/চাকুরী/অন্যান্য
১১. সঙ্গীর (স্বামী বা স্ত্রীর) পেশাঃ ১) গৃহকাজ/চাকুরী প্রার্থী, ২) কৃষিকাজ, ৩) রিক্সা/দিনমজুর, ৪) ব্যবসা/চাকুরী/অন্যান্য
১২. মাসিক আয় (টাকায়)ঃ ___________
১৩. সঙ্গীর (স্বামী বা স্ত্রীর) মাসিক আয় (টাকায়)ঃ ___________
১৪. আপনার ধর্মীয় সম্প্রদায়ঃ ১) মুসলিম, ২) হিন্দু, ৩) খ্রিস্টান, ৪) অন্যান্য___________

Exposure to Marital Violence (α= 0.87)

আপনার স্বামী/স্ত্রী কি কখনও বিবাহ পরবর্তী সময়ে আপনার সাথে নীচের আচরণ করেছিল?

Emotional abuse (α= 0.70)

Mild

১. আপনাকে গালিগালাজ, অপমান বা তিরস্কার করেছিল?
২. আপনাকে অভিশাপ দিয়েছিল বা অমঙ্গল কামনা করেছিল?
৩. আপনার প্রতি রাগ দেখানো বা ঘৃণা প্রকাশ করেছিল?
৪. আপনাকে বাড়ীর বাইরে অন্য কোথায়ও যেতে বাঁধা দিয়েছিল?
৫. বন্ধু-বান্ধব/আত্মীয়দের সাথে দেখা/যোগাযোগে বাঁধা দিয়েছিল?
৬. পড়াশুনায় বা স্কুল/কলেজে/ খেলাধুলায় যেতে বাঁধা দিয়েছিল?

Severe

৭. আপনাকে মারধোর, খুন বা যখম করার কথা বলে হুমকি দিয়েছিল?
৮. আপনার সাথে আর কিছু কি করেছিলো যাতে আপনি খুব ভয় পেয়েছিলেন?
৯. সন্তান/নিকটজনের সাথে এমন কিছু করেছিলো যাতে আপনি খুব ভয় পেয়েছিলেন?

Physical abuse (α= 0.63)

Mild

১. আপনাকে খামচে ধরেছিল বা চড় বা থাপ্পড় মেরেছিল?
২. ধাক্কা দিয়েছিল বা জোরে মুখ/ঠোঁট বা অন্যস্থান চেপে ধরেছিল?

Severe

৩. আপনাকে ঘুসি দিয়েছিল বা লাথি মেরেছিল?
৪. লাঠি, বেত, খুনতি, ঝাটা বা এমন কিছু দিয়ে মেরেছিল?

৫. আপনাকে বালিশ চাপা বা অন্য কোনভাবে দম বন্ধ করেছিল?
৬. আপনাকে সিগারেট বা গরম কিছু বা আগুন দিয়ে ছ্যাকা দিয়েছিল?
৭. আপনাকে লক্ষ্য করে গরম জল, তেল বা অনুরূপ কিছু ছুড়ে মেরেছিল?
৮. ছুরি, দা, বটি, কুড়াল, বন্দুক বা অনুরূপ কিছু দিয়ে আঘাত করেছিল?

Sexual abuse (α= 0.80)

Mild

১. যৌন হয়রানির উদ্দেশ্যে কি কখনো আপনার চুল, হাত, পা, কোমর, পিঠ বা পাছা স্পর্শ করেছিল?
২. ইচ্ছার বিরুদ্ধে পর্ণ ছবি দেখিয়েছিল বা দেখতে বাধ্য করেছিল?
৩. ইচ্ছার বিরুদ্ধে বিবস্ত্র করেছিল বা বিবস্ত্র হতে বাধ্য করেছিল?
৪. ইচ্ছার বিরুদ্ধে অন্য কাউকে বিবস্ত্র অবস্থায় দেখতে বাধ্য করেছিল?
৫. ইচ্ছার বিরুদ্ধে আপনার যৌনাঙ্গ বা লজ্জাস্থান স্পর্শ করেছিল?
৬. ইচ্ছার বিরুদ্ধে অন্যের যৌনাঙ্গ/লজ্জাস্থান স্পর্শ করতে বাধ্য করেছিল?

Severe

৭. আপনার ইচ্ছার বিরুদ্ধে যৌন সঙ্গম করতে বাধ্য করেছিল?
৮. ইচ্ছার বিরুদ্ধে পায়ূ বা মুখ সঙ্গমে অংশ নিতে বাধ্য করেছিল?
৯. জোর করে বা ভয় দেখিয়ে আপনার সাথে যৌন সঙ্গম করেছিল?

স্কোরিং:
বিয়ের পর কখনো ঘটেছে কি? (০) না, (১) হ্যাঁ
বিগত এক বছরে কতবার ঘটেছে? (০) একবারও না, (১) ১-৪ বার, (২) ৫-৯ বার, (৩) ১০ বার বা অধিক

English Version

Socio-demographic Profile

1. Ethnic community: (1) Bengali, (2) Santal, (3) Garo
2. Respondent’s sex: (1) Female, (2) Male
3. Current age in years: __________
4. Spouse’s age in years: __________
5. Length of marriage in years: __________
6. Number of children: __________
7. Family structure: (1)Nuclear, (2) Extended
8. Educational attainment in years: __________
9. Spousal educational attainment in years: __________
10. Main occupation: (1) Home-making/employment seekers, (2) Farming, (3) Day laborers/Rickshaw pullers, (4) Business/job/others__________
11. Spousal occupation: (1) Home-making/employment seekers, (2) Farming, (3) Day laborers/Rickshaw pullers, (4) Business/job/others__________
12. Monthly income (in Taka): __________
13. Spousal monthly income (in Taka): __________
14. Religious community: (1) Muslim, (2) Hindu, (3) Christian, (4) Others

Exposure to Marital Violence (α= 0.87)

Has your husband/wife ever done the following behaviors with you?

Emotional abuse (α= 0.70)

Mild

1. Humiliated, insulted, or shouted at you?
2. Cursed, or wished misfortune for you?
3. Showed anger or expressed hatred at you?
4. Restricted you to go out of home or other places?
5. Restricted you to meet/contact with your parents, relatives, or friends?
6. Restricted you to do your study/attending school/college, or entertaining activities?

Severe

7. Threatened you to kill or injure you seriously?
8. Did something with you so that you got so frightened?
9. Did something with your children/closed ones so that you got so frightened?

Physical abuse (α= 0.63)

Mild

1. Twishted your arm or hair, slapped you?
2. Pushed, slammed, grabbed, or shoved you?

Severe

3. Punched you or kiched you?
4. Beaten up you with stick or something else?
5. Choked you with pillow or other way?
6. Burned you with something hot/fire?
7. Thrown at you hot water/oil/something else?
8. Hitted you with knife or something sharp things?

Sexual abuse (α= 0.80)

Mild

1. Touched your hair, hand, leg, backside, or hip for sexual harrassement?
2. Compelled you to see porn picture/movie against your willingness?
3. Compelled you to undress you against your willingness?
4. Compelled you to see other undressed against your willingness?
5. Touched your sex organs (breast/vagina/penis) against your willingness?
6. Compelled you to touch others' sex organs against your willingness?

Severe

7. Compelled you to participate in sex when you were not interested?
8. Compelled you to participate in anal or oral sex against your willingness?
9. Used physical force and/or intimidation for doing sex with you?

Scoring:
Experienced ever in the post-marital life: (0) No, (1) Yes
Experienced in the last year: (0) none, (1) 1-4 times (2) 5-9 times (3) 10 times/more
