# Supplementary material for: Gender differences in marital violence: A cross-ethnic study among Bengali, Garo, and Santal communities in rural Bangladesh
Source: PLoS One. 2021 May 19;16(5):e0251574. doi: 10.1371/journal.pone.0251574 (PMC8133476; doi:10.1371/journal.pone.0251574)
Supplement: S2 File — (PDF) [file pone.0251574.s005.pdf]

S2 File. List of domestic violence support organizations/networks closed to the study sites

The research team referred the abused respondents seeking help to the following organizations:

<table>
<tr><th>প্রতিষ্ঠানের নাম, ঠিকানা ও ফোন নম্বর</th><th>প্রদত্ত সেবাসমূহ</th></tr>
<tr><td>১. **নারী ও শিশু নির্যাতন প্রতিরোধ হেল্পলাইন সেন্টার: ১০৯**</td><td rowspan="2">• নির্যাতনের শিকার নারী/শিশু, আত্মীয় বা প্রতিবেশী ফোন করে নির্যাতনের কথা জানাতে পারবেন। পরিচয় গোপন রাখা হয় এবং দ্রুত উদ্ধার-টীম পাঠানো হয়।
- নিরাপত্তা ও সকল সেবার ব্যাবস্থা করা হয়।</td></tr>
<tr><td>২. **বাংলাদেশ পুলিশের জরুরিসেবা / হেল্পলাইন নম্বর: ৯৯৯**</td></tr>
<tr><td>৩. **ওয়ান স্টপ ক্রাইসিস সেন্টার/সেলু ওসিসি ***
রাজশাহী মেডিকেল কলেজ হাসপাতাল, মোবাইল: ০১৭১৩ ৩৬৬৬৩৭
টাঙ্গাইল মেডিকেল কলেজ হাসপাতাল, ফোন: ০১৭৪৫ ৪৭৭৭১১, ০১৭৩০ ৭৮১০০৫</td><td>• বিনামূল্যে চিকিৎসা সেবা
• মানসিক সমর্থন ও উপদেশ
• মধ্যস্থতা ও বিরোধ নিস্পত্তি
• বিনামূল্যে আইনগত সহায়তা
• পুলিশি সহায়তা/নিরাপত্তা প্রদান
• নারী/শিশুকে নিরাপদ আশ্রয় প্রদান</td></tr>
<tr><td>৪. **উপজেলা মহিলা বিষয়ক কর্মকর্তার অফিস ***
রাজশাহী, দুর্গাপুর : ফোন: ০১৭১৭-৩৩৯৭৪৯, ০১৭৬২-৮৬৫৬১৬
রাজশাহী, গোদাগাড়ী (উপজেলা পরিষদ): ফোন: ০২৪৭- ৮৫৬২৭৮
টাঙ্গাইল, মধুপুর (উপজেলা পরিষদ): ০১৭১৫৪০৪৭৭৫, ০১৭৬২৬৯১৬৩৬</td><td>• মধ্যস্থতা ও বিরোধ নিস্পত্তি
• বিনামূল্যে আইনগত সহায়তা
• প্রশিক্ষণ ও পুনর্বাসনে সহায়তা</td></tr>
<tr><td>৫. **ব্র্যাক**
রাজশাহী: (গোদাগাড়ী, সি এন বি মোড়), মোবাইল: ০১৭০০-৭৯৬৫৩০
রাজশাহী: (দুর্গাপুর, সিংগা, ,মোবাইল: ০১৭০০-৭৯৬৫২৮
টাঙ্গাইল: (মধুপুর, ময়মনসিংহ রোড), মোবাইল: ০১৭০০-৭৯৬৪২৫</td><td>• মধ্যস্থতা ও বিরোধ নিস্পত্তি
• সাইকো-সোশাল কাউন্সিলিং
• আইনগত সহায়তা</td></tr>
<tr><td>৬. **বাংলাদেশ মহিলা পরিষদ ***
রাজশাহী (রাণীবাজার) , মোবাইল:: ০১৭১২-৮০৩৮৮০
টাঙ্গাইল অফিস (টাঙ্গাইল), মোবাইল: ০১৭১৯-৭৮৬৬১১</td><td>• মধ্যস্থতা ও বিরোধ নিস্পত্তি
• বিনামূল্যে আইনগত সহায়তা
• নারী/শিশুকে নিরাপদ আশ্রয় প্রদান</td></tr>
<tr><td>৭. **বাংলাদেশ লিগ্যাল এইড এবং সেবা ট্রাস্ট (ব্লাস্ট) ***
রাজশাহী: বার এসোসিয়েশন নিউ ভবন, ফোন: ০২৪৭-৮১১৫৩৩, ০১৭৯১-৬৯৪৪৭৯
টাঙ্গাইল: রোকেয়া মঞ্জিল, মেডিকেল কলেজ রোড, ফোন: ০৯২১-৬২২০৭</td><td>• মধ্যস্থতা ও বিরোধ নিস্পত্তি
• বিনামূল্যে আইনগত সহায়তা
• পুলিশি সহায়তা/নিরাপত্তা প্রদান</td></tr>
<tr><td>৮. **বাংলাদেশ জাতীয় মহিলা আইনজীবী সমিতি ***
রাজশাহী: মাছুয়া পাড়ার গলি ,শিরোইল, মোবাইল: ০১৭১১-০৭৫০৬৮
টাঙ্গাইল: বার অফিসের পশ্চিম পাশ, মোবাইল: ০১৮১৮-৪৪৩২৯১</td><td>• মধ্যস্থতা ও বিরোধ নিস্পত্তি
• বিনামূল্যে আইনগত সহায়তা
• নারী/শিশুকে নিরাপদ আশ্রয় প্রদান</td></tr>
<tr><td>৯. **নারী নির্যাতন প্রতিরোধ সেল ***
রাজশাহী: মহিলা বিষয়ক অধিদপ্তর (উপ-পরিচালকের কার্যালয়),
তেরখাদিয়া, ফোন: ০৭২১-৭৬১৭৩৬, ০১৭১৪-২২৯৬৬৬
টাঙ্গাইল: মহিলা বিষয়ক অধিদপ্তর, মোবাইল: ০১৭১৫ ৬০৯১৭৬</td><td>• বিনামূল্যে আইনগত সহায়তা
• প্রশিক্ষণ ও পুনর্বাসনে সহায়তা</td></tr>
<tr><td>১০. **মহিলা সহায়তা কেন্দ্র কর্মসূচি ***
রাজশাহী: বহরমপুর শেষ মাথার মোড়, তেরখাদিয়া পশ্চিম দিক
ফোন: ০৭২১-৭৬১০৬৫, ০১৯১-৬৬৯০৬৯৫</td><td>• মধ্যস্থতা ও বিরোধ নিস্পত্তি
• বিনামূল্যে আইনগত সহায়তা
• নারী/শিশুকে নিরাপদ আশ্রয় প্রদান</td></tr>
<tr><td>১১. **জেলা লিগ্যাল এইড অফিস ***
রাজশাহী, ফোন: ০৭২১- ৭৭১৩৭৩, ০১৭০০-৯৭৯৮৯০, ০১৭০০-৭৮৪২৮৮
টাঙ্গাইল অফিস (টাঙ্গাইল), ফোন: ০৯২১-৬১৬৩৭</td><td>• বিনামূল্যে আইনগত সহায়তা</td></tr>
<tr><td>১২. **এসোসিয়েশন ফর কমিউনিটি ডেভেলপমেন্ট (এসিডি) ***
এইচ-৪১, সাগরপাড়া,৬১০০,রাজশাহী।
ফোন: ০৭২১- ৭৭০৬৬০, ০১৭১৬-৬৮২০৯০</td><td>• মানসিক সমর্থন ও উপদেশ
• বিনামূল্যে আইনগত সহায়তা
• নারী/শিশুকে নিরাপদ আশ্রয় প্রদান</td></tr>
<tr><td>১৩. **সচেতন ***
পলিটেকনিক ইন্সটিটিউট এর পশ্চিমে,সপুরা, রাজশাহী, ফোন: ০১৭৯৩- ০৪০২৭০</td><td>• বিনামূল্যে আইনগত সহায়তা</td></tr>
<tr><td>১৪. **পরিবার ও শিশু কল্যাণ কেন্দ্র ***
মধুপুর: মুক্তিযুদ্ধ অফিসের সামনে ,ময়মনসিংহ রোড, মোবাইল: ০১৭১৩৫৬০৫৮৩</td><td>• মধ্যস্থতা ও বিরোধ নিস্পত্তি
• পরামর্শ ও আইনগত সহায়তা</td></tr>
</table>

*The team had pre-study arrangements with these organizations regarding their referral services

English Version

The research team referred the abused respondents seeking help to the following organizations:

Name of the organization	Given Services
**National Helpline Centre for Violence against Women and Children- 109**	Victim women and children, parents, relatives or neighbor can call to this toll free phone numbers.<br>A law-enforcement team/police take immediate action.<br>– Immediately rescue violence victim women and children and help them.
**National Emergency Service-999**	
**One Stop Crisis Center (OCC)**<br>*Rajshahi Medical College Hospital,* Mobi:01713-366637<br>*Tangail Medical College Hospital,* Phn:01745-477711,01730-781005	– Free medical service<br>– Mental support and advice<br>– Arbitration and dispute mitigation.<br>– Police protection and help; Free Legal Service<br>– Provide secure shelter to victim women and children.
**Upzilla Women Affairs Office**<br>*Rajshahi (Durgapur): Phn:* 01717-339749,01762-865616; Rajshahi *(Godagari), Phn:*0247-856278; *Tangail (Modhupur), Phn:* 01715-404775	– Free legal aid.<br>– Arbitration and dispute mitigation.<br>– Help in rehabilitation and training.
**BRAC**<br>*Rajshahi (Godaghari), Mob:*01700-796530<br>*Rajshahi(Durgapur), Phn:* 01700-796528<br>*Tangail (Modhupur),Phn:*01700- 796425	– Arbitration and dispute mitigation.<br>– legal aid<br>– Psycho-social concealing.
**Bangladesh Mohila Porishad**<br>*Rajshahi (Rani Bazar), Phn:* 01712-803880<br>*Tangail, Phn:* 01719-786611	– Arbitration and dispute mitigation.<br>– Free legal aid<br>– Provide secure shelter to women and children.
**Bangladesh Legal Aid and Service Trust (BLAST),** *Rajshahi (Bar Association New Bhavan), Phn:*0247-811533,01791694479, *Tangail (Rokeya Monjil, Medical College Road), Phn***:**0921-62207	– Arbitration and dispute mitigation.<br>– Free legal aid<br>– Police protection/security.
**Bangladesh National Women Lawyers' Association,** *Rajshahi :(Shiroil) Phn:* 01711-075068, *Tangail (Bar Office), Phn:*01818443291	– Arbitration and dispute mitigation.<br>– Free legal aid<br>– Provide secure shelter to women and children.
**Cell for the prevention of violence against women,** *Department of women affairs*<br>*Rajshahi, Phn:* 0721-761736, 01714-229666<br>*Tangail, Phn:* 01715-609176	– Free legal aid<br>– Help in rehabilitation and training.
**Women's Help Center Programme**<br>***Ministry of women's Affairs,*** *Rajshahi, Mob:* 0721-761065, 01916-690695	– Arbitration and dispute solving.<br>– Free legal aid<br>– Provide secure shelter to women and children.
**District Legal Aid Office**<br>*Rajshahi, phn:*0721-771373, 01700-784288,01700-979890<br>*Tangail, phn:* 0921-61637	– Free legal aid
**Association for Community Development (ACD),**<br>*Rajshahi, Phn:* 0721-770660, 01716-682090	– Mental support and advice<br>– Free legal aid<br>– Provide secure shelter to women and children.
**Socheton**<br>*Rajshahi, Phn:*01793-040270	– Free legal aid.
**Family and Children Welfare Center**<br>*Tangail (Modhupur),Phn:* 01713560583	– Arbitration and dispute mitigation.<br>– Legal aid and advice
